# Reducing HIV infection in people who inject drugs is impossible without targeting recently-infected subjects

**DOI:** 10.1097/QAD.0000000000001291

**Published:** 2016-11-08

**Authors:** Tetyana I. Vasylyeva, Samuel R. Friedman, Jose Lourenco, Sunetra Gupta, Angelos Hatzakis, Oliver G. Pybus, Aris Katzourakis, Pavlo Smyrnov, Timokratis Karamitros, Dimitrios Paraskevis, Gkikas Magiorkinis

**Affiliations:** a Department of Zoology, University of Oxford, Oxford, United Kingdom; b National Development and Research Institutes, Inc, New York, New York, USA; c Department of Hygiene, Epidemiology, and Medical Statistics, Athens University Medical School, Athens, Greece; d International HIV/AIDS Alliance in Ukraine, Kyiv, Ukraine.

**Keywords:** epidemiology, harm reduction, HIV, people who inject drugs, phylodynamics, recent infection

## Abstract

**Objective:**

Although our understanding of viral transmission among people who inject drugs (PWID) has improved, we still know little about when and how many times each injector transmits HIV throughout the duration of infection. We describe HIV dynamics in PWID to evaluate which preventive strategies can be efficient.

**Design:**

Due to the notably scarce interventions, HIV-1 spread explosively in Russia and Ukraine in 1990s. By studying this epidemic between 1995 and 2005, we characterized naturally occurring transmission dynamics of HIV among PWID.

**Method:**

We combined publicly available HIV *pol* and *env* sequences with prevalence estimates from Russia and Ukraine under an evolutionary epidemiology framework to characterize HIV transmissibility between PWID. We then constructed compartmental models to simulate HIV spread among PWID.

**Results:**

In the absence of interventions, each injector transmits on average to 10 others. Half of the transmissions take place within 1 month after primary infection, suggesting that the epidemic will expand even after blocking all the post–first month transmissions. Primary prevention can realistically target the first month of infection, and we show that it is very efficient to control the spread of HIV-1 in PWID. Treating acutely infected on top of primary prevention is notably effective.

**Conclusion:**

As a large proportion of transmissions among PWID occur within 1 month after infection, reducing and delaying transmissions through scale-up of harm reduction programmes should always form the backbone of HIV control strategies in PWID. Growing PWID populations in the developing world, where primary prevention is scarce, constitutes a public health time bomb.

## Introduction

HIV in people who inject drugs (PWID) has, to date, posed a secondary burden worldwide after sexual transmission, with the striking exceptions of eastern European and central Asian countries [1]. Much work has been done to study how HIV spreads in PWID, and now we have interventions that proved to be effective to slow down the HIV epidemic in this group [2]. Still, research to date has not measured forward transmission (i.e. number of transmissions that can be attributed to an infected person), and our knowledge is limited to the force of infection (i.e. the probability of PWID getting infected over time) [3].

With almost 1 million HIV-infected individuals and a PWID population of around 2 million people [1], Russia and Ukraine provide a unique opportunity for studying HIV transmission dynamics in this group. The epidemic in these countries has been accompanied by very limited harm reduction services [4], making it a large-scale ‘natural experiment’ of HIV spread among PWID. By studying the epidemiological dynamics of the largest HIV epidemic among PWID, we might understand epidemics emerging in countries of the Middle East and northern Africa [5,6], southeastern Asia [7], Europe [8,9], and the USA [10]. Critically, although harm reduction approaches among PWID since the 1980s have had significant effects in the developed world [11], it remains unclear whether these preventive strategies should be prioritized over newer approaches such as treatment as prevention (TasP) [12] in resource-limited settings.

Here, we combined the evolutionary epidemiology approach with simple mathematical modelling to describe transmission dynamics of the HIV epidemic among PWID in Ukraine and Russia that started in the mid-1990s and assess factors mitigating HIV epidemics in PWID.

## Methods

### Nucleotide sequences

We compiled three HIV-1 sequence alignment datasets with sequences available from the Los Alamos HIV sequence database (http//:hiv.lanl.gov) (Supplementary Table 2): a *reference dataset A* of 2199 *pol* sequences; *dataset B* with 418 *pol* sequences from Russia and Ukraine sampled in 1997–2013; *dataset C* with 92 *env* sequences from Russia and Ukraine sampled in 1993–2011. All sequence alignments were performed using MEGA 6.0 software [13] and then manually edited. We built Bayesian Skyline Plots with BEAST 1.8.1 [14,15].

### Estimates of epidemiological parameters

To obtain estimations of some of the epidemiological parameters, we used our previously described methods [16]. We estimated the number of secondary infections per primary infection in a completely susceptible population as
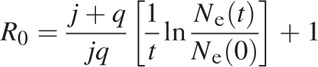


where *N*_e_(*t*) is the effective population size at time *t*, *N*(0) is the effective population size at the baseline of the exponential growth phase, *j* is the rate of progression from the recent to long-term infection (1/0.5 a year, 2.0), and *q* is the overall mortality rate of HIV-infected individuals (in the absence of prevention assumed to average 1/10 years, 0.1).

We assume that *R*_0,a_ is the mean number of secondary infections per transmitter (per injector) for our epidemiological settings:
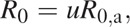


where *u* is the proportion of PWID in the HIV-infected population.

We estimate the generation time *T* (the expected time from initial HIV infection to transmission to other individuals) as
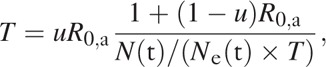


where *N*_e_(t) × *T* is estimated from the Bayesian Skyline Plot reconstructed from the HIV sequences (*env* gene).

### Compartmental modelling

We used a compartmental Susceptible – Recently Infected – Chronically Infected (Susceptibles – individuals who are not infected, but are at risk of infection; Recently Infected – individuals who acquired infection within the last 6 months; and Chronically Infected – individuals who acquired infection longer than 6 months ago) model to describe dynamics of HIV epidemic among PWID in Russia and Ukraine (Supplementary Fig. 1). We further advanced this model to include the compartment of treated individuals and take into account the uncertainty of our estimates.

More details on the methods can be found in Supplementary text. A description of the model parameters is provided in Supplementary Table 1.

## Results

### Phylodynamics and epidemiologic parameters estimates

**Fig. 1:**
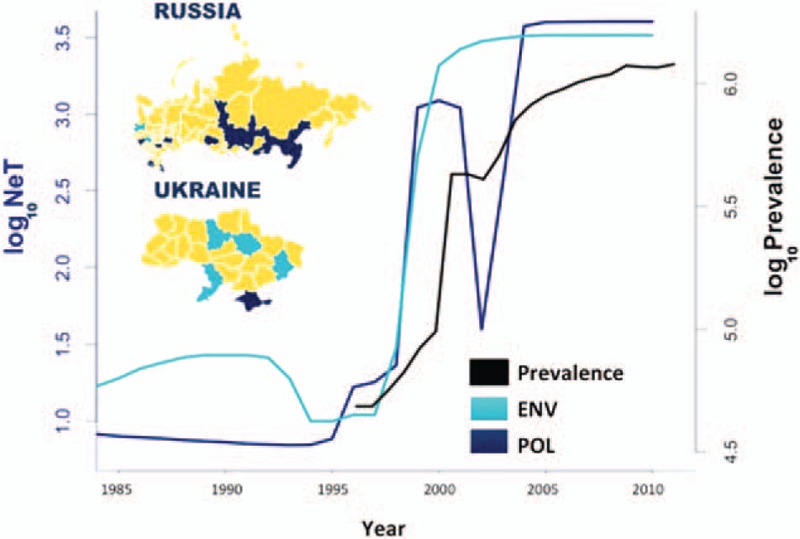
Bayesian Skyline Plot estimated from pol (dark blue line) and env (cyan line).

The Bayesian Skyline Plots of the HIV population in Ukraine and Russia (Fig. 1) estimated from the *pol* and *env* alignments closely followed the epidemiological estimates of HIV prevalent cases: the epidemic grew rapidly between 1995 and 2000, and slower growth continued until 2005. Using the skyline plot and the estimated number of prevalent cases, we calculated *R*_0,a_ to be 6–15 for corresponding values of the duration of infectivity (*p*) 5–15 years (Supplementary Fig. 2A). A plausible duration of infectivity for untreated HIV-infected PWID is unlikely to be more than 8–10 years [17], suggesting that the epidemic in Russia and Ukraine had an *R*_0,a_ of 8–10. We calculated *T* using different values of the proportion of transmitters, *u*, approximated by the proportion of PWID among the HIV infected population. *T* was estimated to be 25 days when *R*_0,a_ is around 10 and *u* = 70% (Supplementary Fig. 2B); *T* would exceed 3 months if *u* was less than 30%.

Maps show representation of sequences by geographical regions within the countries. The UNAIDS estimates of HIV prevalent cases are shown by the black line (prevalence).

### Modelling the establishment of HIV epidemics in people who inject drugs

In the natural epidemic model without treatment or prevention, HIV prevalence among PWID was predicted to reach 86% [credibility interval (CI) 81–88%] after 20 years (Supplementary Fig. 3). The proportion of transmissions attributed to recently infected individuals rapidly decreased over the first years of the epidemic and stabilized at around 20% after 7 years (Supplementary Fig. 4).

**Fig. 2:**
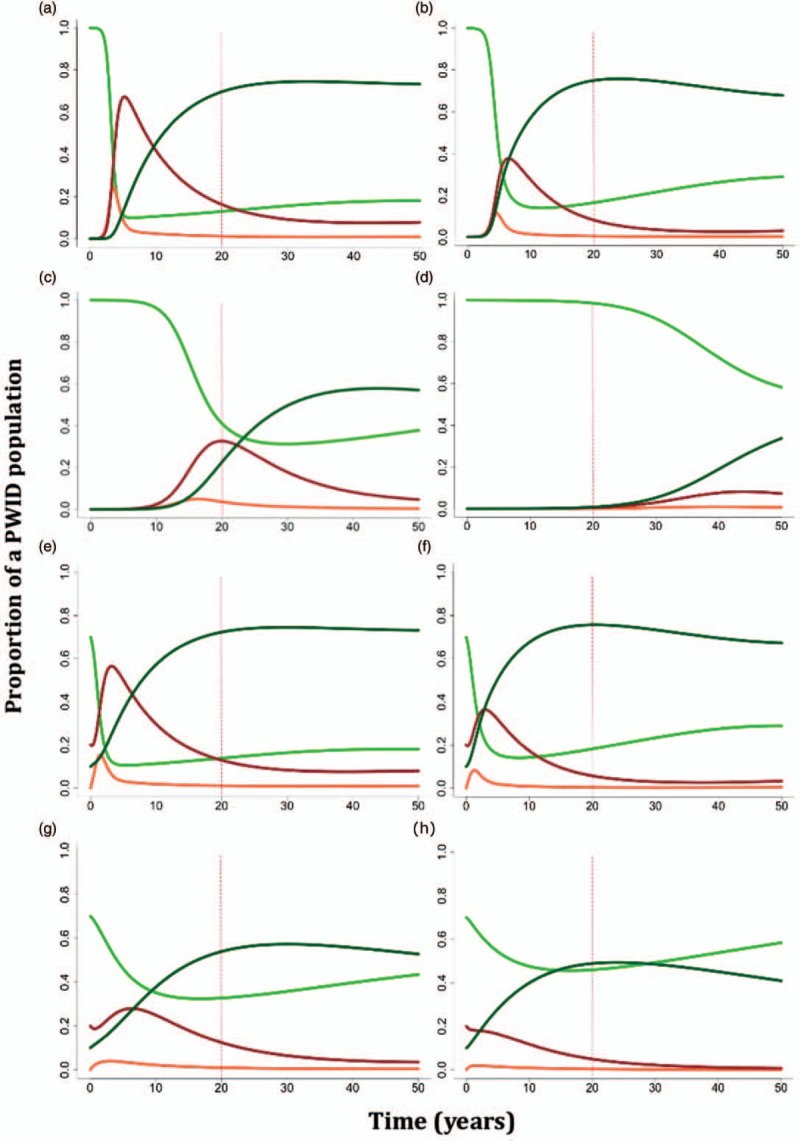
Results of the compartmental model representing HIV spread under different treatment scenarios.

In absence of prevention, but with treatment, the model showed that HIV prevalence would still reach 83% (CI 73–87%) after 20 years if 50% of long-term infected individuals receive treatment on average 4 years after becoming infected (Fig. 2a). If treatment was provided to a small proportion (25%) of recently infected individuals, after 20 years we did not observe a dramatic reduction in prevalence (72%, CI 62–80%) (Fig. 2b). We next investigated the scenario where *R*_0_ was reduced from the very beginning of an outbreak by scaling up harm reduction interventions and changing risky behaviours. By reducing *R*_0,a_ by 60% (studies show that harm reduction programmes can help to reduce risky practices by 60% [18,19] and incidence in PWID by 80% [20]) to *R*_0,a_ = 4, we were able to prevent the epidemic from establishing, provided that treatment was offered to at least 25% of recently infected PWID (Fig. 2d). There was no intervention scenario to prevent the epidemic from being established when treatment was not provided to recently infected individuals (Fig. 2c). For the scenarios with the well established epidemic (30% baseline HIV prevalence) (Fig. 2e–h), the best case scenario was also to treat at least a small proportion of recently infected individuals while scaling up prevention.

(a)–(d) HIV spread during an outbreak. (a) and (b) Scenarios when 0 and 25% of recently infected individuals, respectively, and 50% of long-term infected individuals (for both a and b) are provided with treatment, while no other preventive services are suggested. (c) and (d) Scenarios when, in addition to providing treatment, other harm reduction interventions are introduced, so that *R*_0,a_ is reduced to 4. (e)–(h) HIV spread in a well established epidemic. (e) and (f) Scenarios when 0 and 25% of recently infected individuals, respectively, and 50% of long-term infected individuals (for both e and f) are provided with treatment, while no other preventive services are suggested. (g) and (h) Scenarios when, in addition to providing treatment, other harm reduction interventions are introduced, so that *R*_0,a_ is reduced to 4.

## Discussion

We analysed the uncontrolled and extensive HIV epidemic in Russia and Ukraine that began in the mid-1990s with an evolutionary epidemiology approach. We estimated that at the time, each injector transmitted to at least five individuals within the first month after getting infected. We thus showed that in the absence of prevention, HIV transmissions in PWID occur rapidly after primary infection.

Can we realistically target the first month of the infection? With primary prevention, such as syringe exchange programmes (SEPs) and safe practice training, we intervene from the very first day of infections. As the infectivity drops after the first month as a result of lower viral load and established immune responses, if we were to increase generation time, we would reduce the probability of infection per contact; we would then have more time to intervene with treatment.

Indeed, we show that TasP has a place in controlling an outbreak only when we introduce large-scale primary prevention and appeared to be more effective when we targeted recently infected individuals. Other studies show the importance of recently infected individuals in sexually driven HIV epidemics [21–23], but no consensus was reached on whether aiming HIV prevention efforts specifically at the recently infected group would be game-changing [24,25]. While such evidence for PWID is largely missing, we show that prevention of early transmissions provides major gains when we reduce transmissibility by reducing risky injecting practices.

Our findings provide insights on the dynamics of recently emerging HIV outbreaks such as those in Athens [8], Romania [9], or Indiana [26]. Crucially, HIV epidemics in PWID have been recently emerging in resource-limited countries in the Middle East and north Africa (particularly Afghanistan, Egypt, and Morocco) [5], as well as in some southeast Asian countries (e.g. Indonesia and Philippines) [7,27]. PWID populations in these locations were historically small, but their growth created the potential for new HIV outbreaks. Our study suggests that, if primary prevention is not introduced in countries with increasing prevalence of drug use, the epidemics in this group will expand rapidly when started and remain uncontrolled until sufficient primary prevention is put in place. Given that intravenous drug use is increasing faster than preventive responses in the developing world, it is a matter of time that these public health time bombs will explode again.

Changes in drug-taking behaviour might also influence transmission dynamics and introduce new pools of susceptible individuals within PWID populations. In our results, the drop of *N*_e_*T* around 2002 might be partly attributed to the shift in the Russian–Ukrainian drug scene towards stimulant drugs (associated with risky injecting behaviours [28]). Thus, settings with changing drug scenes, including changes in drug of choice or demographic changes, should also be addressed with basic prevention scale-up.

Our modelling suggested that increasing SEP coverage and reducing risky injecting practices in addition to treatment provision to half of the infected PWID will yield an additional 15% decrease in HIV prevalence after 20 years. Another modelling study that was informed by data from Ukraine showed that a 40% reduction in HIV prevalence within 10 years period should be expected if at least 60% of the unmet needs of harm reduction services (including SEPs and opioid substitution treatment) and treatment were covered [29], but this study did not model differences in transmissibility among recently and chronically infected. Our modelling suggested that such a steep decrease in HIV prevalence can be obtained only if we additionally provide at least 25% of recently infected individuals with treatment.

Apart from epidemiological reasons, early treatment initiation provides clear benefits for the patients themselves [30,31]. Thus, a combination of the expansion of existing harm reduction interventions and the development of new approaches to identify and treat recently infected people should be applied to settings with PWID-driven HIV epidemics [32]. Unfortunately, reaching those recently infected is extremely challenging, especially in settings in which preventive responses have been limited due to the sociopolitical context in which the outbreak arises. In the 1990s, social stigmatization of PWID in Russia and Ukraine was reflected in governmental and healthcare sectors, which limited the implementation of harm reduction services [4,33]. Other developing countries experiencing HIV outbreaks among PWID face similar problems; for example, despite decriminalization of drug use, police harassment of PWID in Indonesia undermines the success of SEPs [34]. Political support to reduce stigmatization of PWID could significantly increase access to health services and mitigate HIV spread in this vulnerable group.

## Acknowledgements

T.I.V. and G.M. developed the research question, T.I.V., G.M., J.L., and T.K. contributed to the data analysis. T.I.V., S.R.F., J.L., S.G., A.H., O.G.P., A.K., P.S., T.K., D.P., and G.M. wrote the manuscript. T.I.V. is supported by the Clarendon Fund and Hertford College of the University of Oxford, S.R.F. is supported by the NIH NIDA (Grant number DP1DA034989). G.M. is supported by an MRC Clinician Scientist Fellowship (MR/K010565/1).

### Conflicts of interest

There are no conflicts of interest.

## Supplementary Material

Supplemental Digital Content

## References

[1] Joint United Nations Programme on HIV/AIDS. UNAIDS report 2014: the Gap report. Geneva, Switzerland: Joint United Nations Programme on HIV/AIDS; 2014.

[2] MacArthur GJ van Velzen E Palmateer N Kimber J Pharris A Hope V Interventions to prevent HIV and hepatitis C in people who inject drugs: a review of reviews to assess evidence of effectiveness. *Int J Drug Policy* 2014; 25:34–52. 23973009 10.1016/j.drugpo.2013.07.001

[3] Thomas MSD Raj Y Pandey A A probability model for estimating the force of transmission of HIV infection and its application. *Am J Math Stat* 2014; 4:171–177.

[4] Harm Reduction International. The global state of Harm Reduction. Towards an integrated response. In. Edited by Stoicescu C. London: Harm Reduction International; 2012.

[5] Mumtaz GR Weiss HA Thomas SL Riome S Setayesh H Riedner G HIV among people who inject drugs in the Middle East and North Africa: systematic review and data synthesis. *PLoS Med* 2014; 11:e1001663. 24937136 10.1371/journal.pmed.1001663 PMC4061009

[6] Ghasemian A Mostafavi SKS Vardanjani A-RK Human immunodeficiency virus (HIV) distribution in Middle East region. *Avicenna J Clin Microb Infect* 2016; 3:e35965.

[7] Afriandi I Aditama TY Mustikawati D Oktavia M Alisjahbana B Riono P HIV and injecting drug use in Indonesia: epidemiology and national response. *Acta Med Indones* 2009; 41 suppl 1:75–78. 19920303

[8] Paraskevis D Nikolopoulos G Tsiara C Paraskeva D Antoniadou A Lazanas M HIV-1 outbreak among injecting drug users in Greece, 2011: a preliminary report. *Euro Surveill* 2011; 16:16. 10.2807/ese.16.36.19962-en 21924120

[9] Niculescu I Paraschiv S Paraskevis D Abagiu A Batan I Banica L Recent HIV-1 outbreak among intravenous drug users in Romania: evidence for cocirculation of CRF14_BG and subtype F1 strains. *AIDS Res Hum Retroviruses* 2014; 31:488–495. 25369079 10.1089/aid.2014.0189 PMC4426324

[10] Conrad C Bradley HM Broz D Buddhal S Chapman EL Galang RR Community outbreak of HIV infection linked to injection drug use of oxymorphone – Indiana, 2015. *MMWR Morb Mortal Wkly Rep* 2015; 64:443–444. 25928470 PMC4584812

[11] Wodak A McLeod L The role of harm reduction in controlling HIV among injecting drug users. *AIDS* 2008; 22 suppl 2:S81–92. 10.1097/01.aids.0000327439.20914.33 PMC3329723 18641473

[12] Cohen MS Chen YQ McCauley M Gamble T Hosseinipour MC Kumarasamy N Prevention of HIV-1 infection with early antiretroviral therapy. *N Engl J Med* 2011; 365:493–505. 21767103 10.1056/NEJMoa1105243 PMC3200068

[13] Tamura K Stecher G Peterson D Filipski A Kumar S MEGA6: molecular evolutionary genetics analysis version 6.0. *Mol Biol Evol* 2013; 30:2725–2729. 24132122 10.1093/molbev/mst197 PMC3840312

[14] Drummond AJ Rambaut A BEAST: Bayesian evolutionary analysis by sampling trees. *BMC Evol Biol* 2007; 7:214. 17996036 10.1186/1471-2148-7-214 PMC2247476

[15] Drummond AJ Ho SY Phillips MJ Rambaut A Relaxed phylogenetics and dating with confidence. *PLoS Biol* 2006; 4:e88. 16683862 10.1371/journal.pbio.0040088 PMC1395354

[16] Magiorkinis G Sypsa V Magiorkinis E Paraskevis D Katsoulidou A Belshaw R Integrating phylodynamics and epidemiology to estimate transmission diversity in viral epidemics. *PLoS Comput Biol* 2013; 9:e1002876. 23382662 10.1371/journal.pcbi.1002876 PMC3561042

[17] Longini IM Jr Clark WS Byers RH Ward JW Darrow WW Lemp GF Statistical analysis of the stages of HIV infection using a Markov model. *Stat Med* 1989; 8:831–843. 2772443 10.1002/sim.4780080708

[18] Huo D Ouellet LJ Needle exchange and injection-related risk behaviors in Chicago: a longitudinal study. *J Acquir Immune Defic Syndr* 2007; 45:108–114. 17460474 10.1097/QAI.0b013e318050d260

[19] Bluthenthal RN Kral AH Gee L Erringer EA Edlin BR The effect of syringe exchange use on high-risk injection drug users: a cohort study. *AIDS* 2000; 14:605–611. 10780722 10.1097/00002030-200003310-00015

[20] Des Jarlais DC Marmor M Friedmann P Titus S Aviles E Deren S HIV incidence among injection drug users in New York City, 1992–1997: evidence for a declining epidemic. *Am J Public Health* 2000; 90:352–359. 10705851 10.2105/ajph.90.3.352 PMC1446171

[21] Brenner BG Roger M Routy JP Moisi D Ntemgwa M Matte C High rates of forward transmission events after acute/early HIV-1 infection. *J Infect Dis* 2007; 195:951–959. 17330784 10.1086/512088

[22] Powers K Ghani A Miller W Hoffman I Pettifor A Hosseinipour M The contribution of HIV-discordant couples to HIV transmission in Lilongwe, Malawi. *J Int AIDS Soc* 2012; 15:79–179.

[23] Pinkerton SD How many sexually-acquired HIV infections in the USA are due to acute-phase HIV transmission?. *AIDS* 2007; 21:1625–1629. 17630558 10.1097/QAD.0b013e32826fb6a6 PMC2377417

[24] Eaton JW Hallett TB Why the proportion of transmission during early-stage HIV infection does not predict the long-term impact of treatment on HIV incidence. *Proc Natl Acad Sci U S A* 2014; 111:16202–16207. 25313068 10.1073/pnas.1323007111 PMC4234601

[25] Vasylyeva TI Friedman SR Magiorkinis G Prevention of early HIV transmissions might be more important in emerging or generalizing epidemics. *Proc Natl Acad Sci U S A* 2015; 112:E1515. 25737538 10.1073/pnas.1424168112 PMC4386368

[26] Rich JD Adashi EY Ideological anachronism involving needle and syringe exchange programs: lessons from the Indiana HIV outbreak. *JAMA* 2015; 314:23–24. 26000661 10.1001/jama.2015.6303 PMC4496270

[27] Farr AC Wilson DP An HIV epidemic is ready to emerge in the Philippines. *J Int AIDS Soc* 2010; 13:16. 20409346 10.1186/1758-2652-13-16 PMC2868805

[28] Booth RE Lehman WE Kwiatkowski CF Brewster JT Sinitsyna L Dvoryak S Stimulant injectors in Ukraine: the next wave of the epidemic?. *AIDS Behav* 2008; 12:652–661. 18264752 10.1007/s10461-008-9359-3

[29] Strathdee SA Hallett TB Bobrova N Rhodes T Booth R Abdool R HIV and risk environment for injecting drug users: the past, present, and future. *Lancet* 2010; 376:268–284. 20650523 10.1016/S0140-6736(10)60743-X PMC6464374

[30] Group TAS Danel C Moh R Gabillard D Badje A Le Carrou J A trial of early antiretrovirals and isoniazid preventive therapy in Africa. *N Engl J Med* 2015; 373:808–822. 26193126 10.1056/NEJMoa1507198

[31] Group ISS Lundgren JD Babiker AG Gordin F Emery S Grund B Initiation of antiretroviral therapy in early asymptomatic HIV infection. *N Engl J Med* 2015; 373:795–807. 26192873 10.1056/NEJMoa1506816 PMC4569751

[32] Vasylyeva TI Friedman SR Smyrnov P Bondarenko K A new approach to prevent HIV transmission: project protect intervention for recently infected individuals. *AIDS Care* 2015; 27:223–228. 25244688 10.1080/09540121.2014.947913 PMC4524562

[33] Csete J Human Rights Watch (Organization). Lessons not learned: human rights abuses and HIV/AIDS in the Russian Federation. New York, NY: Human Rights Watch; 2004.

[34] Mboi N Indonesia. A consummate insider pushes ideas from outside Indonesia. *Science* 2014; 345:162–163. 25013063 10.1126/science.345.6193.162

