# Supplementary material for: Reducing HIV infection in people who inject drugs is impossible without targeting recently-infected subjects
Source: AIDS. 2016 Nov 8;30(18):2885–90. doi: 10.1097/QAD.0000000000001291 (PMC5106086; doi:10.1097/QAD.0000000000001291)
Supplement: Supplemental Digital Content [file aids-30-2885-s001.docx]

**Supplementary Text**

**Mitigating HIV in People Who Inject Drugs is impossible without targeting recently-infected: what we learned from Russia and Ukraine**

Tetyana I VASYLYEVA^a^, MS, Samuel R FRIEDMAN^b^, PhD, Jose LOURENCO^a^, PhD, Sunetra GUPTA^a^, PhD, Angelos HATZAKIS^c^, PhD, Oliver G PYBUS^a^, DPhil, Aris KATZOURAKIS^a^, PhD, Pavlo SMYRNOV^d^, MS, Timokratis KARAMITROS^a,c^, PhD, Dimitrios PARASKEVIS^c^, PhD, Gkikas MAGIORKINIS, PhD^a, *^

a – Department of Zoology, University of Oxford, South Parks Road, OX1 3PS, Oxford, United Kingdom

b - National Development and Research Institutes, Inc, New York, New York 10010, USA

c - Department of Hygiene, Epidemiology, and Medical Statistics, Athens University Medical School, 75, M. Asias Street, Athens 115 27, Greece

d – International HIV/AIDS Alliance in Ukraine, 5 Dilova str., Kyiv 03680, Ukraine

*To whom correspondence should be addressed:

Dr. Gkikas Magiorkinis, MD, PhD, FRCPath

South Parks Road,

OX1 3PS,

Oxford,

United Kingdom

[gkikas.magiorkinis@zoo.ox.ac.uk](mailto:gkikas.magiorkinis@zoo.ox.ac.uk)

**Table of Contents Page**

PHYLOGENETICS…………………………………………………………………..3

Nucleotide sequences…………………………………………………………3

Phylogenetic analysis…………………………………………………………4

ESTIMATES OF EPIDEMIOLOGICAL PARAMETERS…………………….…….4

Parameters derivations……………………………………………………...…4

Results and interpretations of epidemiological parameters calculations……...6

COMPARTMENTAL MODELLING……………………………………..….………6

Model description……………………………………………...………………6

SENSITIVITY AND LIMITATIONS……………………………….…………..……9

Sensitivity analyses………………………………………………………...….9

Limitations-Assumptions on network effects……………………………......10

SUPPLEMENTARY FIGURES………………….………………………………….11

Suppl. Fig.1……………….………………………………………………….11

Suppl. Fig.2……………….………………………………………………….12

Suppl. Fig.3……………….………………………………………………….13

Suppl. Fig.4……………….………………………………………………….14

Suppl. Fig.5……………….………………………………………………….15

Suppl. Fig.6……………….………………………………………………….16

Suppl. Fig.7……………….………………………………………………….17

REFERENCES……………………………….…………………………………...….18Supplementary Table 1……………….………………………………………………20

Supplementary Table 2……………….………………………………………………22

**PHYLOGENETICS**

**Nucleotide sequences**

We compiled three HIV-1 sequence alignment datasets from the Los Alamos HIV sequence database (http//:hiv.lanl.gov). Firstly, a *reference dataset A* of 2199 (all available) sequences of minimal length of 300 nucleotides (nt) from the *pol* region of HIV genome (nt 6225-8795) accessed in 2011 from HIV sequence database (sequence names are in the Suppl. Table 2). Sequences came from 36 countries (Afghanistan, Albania, Azerbaijan, Burkina Faso, Burundi, Benin, Democratic Republic of Congo, Chad, Congo, Cameroon, Cuba, Cyprus, Czech Republic, Ethiopia, France, Gabon, Georgia, Ghana, Equatorial Guinea, Kenya, Kazakhstan, Latvia, Mali, Nigeria, Russia, Rwanda, Sudan, Slovenia, Senegal, Togo, Tanzania, Ukraine, Uganda, Uzbekistan) sampled in 1985-2010. We aligned the sequences using MEGA 6.0 software [1] and then manually edited. We constructed phylogenetic trees with Mega 6.0 with simple evolutionary models to reduce computational complexity (i.e. assuming uniform rates among sites, using neighbour-joining approach with Tamura-Nei model and same parameters were kept for other phylogenetic trees constructed during these analyses). Interestingly, the strains that were isolated from countries of the former Eastern block clustered together with high bootstrap support (>0.9). Given that previously published data suggested that the epidemic in Russia and Ukraine started in Odessa, Ukraine and then expanded through the whole region [2, 3], the following analysis was conducted for sequences from Russia and Ukraine together as coming from the same HIV epidemic. Consequently, all sequences from Russia and Ukraine from the *pol* region were sub-sampled from dataset A and sequences available from later years were added. This formed *dataset B* with 418 *pol* sequences from Russia and Ukraine sampled in 1997-2013.

Further, 92 sequences from Russia and Ukraine from the *env* region sampled in 1993-2011 were downloaded from the same database and formed the *dataset C*. Alignments were performed in Mega 6.0 and manually edited. Only one sequence from each patient was included: if there was evidence that two sequences come from the same person (based on the sequence names or the absence of genetic distance between two sequences), one of them was deleted from the dataset. Accession numbers of all sequences are in the Suppl. Table 2.

Both of the phylogenetic trees (constructed with *env* and *pol* alignments) suggested that the subtype A epidemic in Russia and Ukraine was introduced once for both of these countries and thus allowed us to run all further analyses considering them to be part of the same epidemic.

# We run population dynamics analyses using Bayesian Evolutionary Analysis Sampling Trees (BEAST) 1.8.1 software [4]. Initially, we analysed dataset C (*env*) creating a subgroup of sequences that had both *env* and *pol* genes. We estimated the time to most recent common ancestor (TMRCA) for this group and used it as a prior on TMRCA to inform the analysis of dataset B (*pol*). We used the GTR nt substitution model and assumed sites heterogeneity with Gamma distribution with 4 invariant sites. We applied uncorrelated relaxed clock model to reconstruct population dynamics [5], and subsequently constructed a Bayesian Skyline plot to get the product of an effective population size (*N*_e_, the size of an idealized population that has the same population dynamic properties as actual population of interest) and a generation time (*T*, the expected time from acquiring infection to onward transmission) and construct the coalescent phylogenetic tree.

We ran a Markov chain Monte Carlo (MCMC) for the datasets B and C separately: 100,000,000 generations for the dataset C with *env* sequences (burn-in 20 x 10^6^ generations), and two separate 100,000,000 generations runs for the dataset C with *pol* sequences (burn-in 20 x 10^6^ and 40 x 10^6^ generations). Trees were sampled every 1000 generations. We used the LogCombiner program from the BEAST 1.8.1 package to combine the two runs from *pol* region, and the program Tracer from the same package to check for convergence and determine whether appropriate mixing of the MCMC sampler had been achieved in the posterior target distribution (effective sample size>100).

We obtained yearly estimates of HIV prevalent cases for all the available years (1990-2011) from the UNAIDS website [6].

**Phylogenetic analysis**

The phylogenetic tree, which included globally representative subtype A reference strains, shows that the strains from the epidemic in Russia, Ukraine and other countries of the former Eastern bloc formed a distinct clade within the global HIV-1 epidemic (Suppl.Fig.5). This supports the previous report that an introduction of HIV-1 subtype A to Odessa, Ukraine, seeded the epidemic in Ukraine and Russia [2, 3], suggesting that the Russian and Ukrainian epidemics were isolated from the rest of the world but spread as a joint epidemic. We, thus integrated molecular and epidemiological data from these areas and analysed them as a single epidemic.

The skyline plot estimated from the *pol* sequences showed a dramatic drop and rebound in *N*_e_*×T* around 2002-03 (Fig.1). The drop was significant given the 95% highest posterior density (HPD) intervals (Suppl.Fig.6) and appeared in Skyline plots reconstructed from all three random sub-samples of *pol* sequences (data not shown); it was not, however, apparent in the skyline plot estimated from *env* sequences. We concluded that the discordance among *env* and *pol* skylines could be attributed to their geographic distribution, with sequences from *pol* being more representative of the Russian and Ukrainian PWID population than sequences from *env* (19 vs 7 geographic regions, respectively, see map on Fig.1).

We estimated the *PTP* to be 421.5 (95% CI 248.6-594.4) through a linear regression of the estimated number of prevalent cases for Ukraine and Russia over the median *N*_e_*×T*. We then used *PTP* together with the changes in the effective population size to calculate the *R*_0_ and generation time *T* as described below and previously reported [7].

**ESTIMATES OF EPIDEMIOLOGICAL PARAMETERS**

**Parameters derivations**

We estimated the number of secondary infections per primary infection in a completely susceptible population assuming that the population is large enough to follow a deterministic Susceptible-Infected-Removed model [8]:

(1)
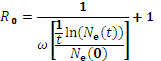

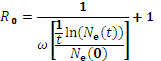


where *N*_e_(*t*) is the effective population size at time t (*N*_e_*×T* was estimated from the skyline plot reconstructed from *env* sequences), *N*_e_(0) is the effective population size at the baseline of the exponential growth phase, and *ω* is the average removal rate.

For our model we estimated the average removal rate as:

(2)
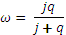

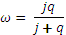


where *j* is the rate of progression from the recent to long-term phase of infection (1/0.5 a year, 2.0), *q* is the overall mortality rate of HIV infected individuals (in the absence of prevention assumed to average 1/10 years, 0.1). Thus, the number of secondary infections per primary infection in our model is described as:

(3)
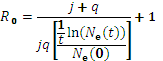

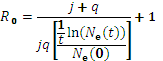


Further, *N*(*t*) is the estimate of HIV prevalence at time *t*. We obtained yearly HIV number of prevalent cases estimates for all the available years (1990-2011) from the UNAIDS website [6]. The ratio *N*(*t*)/*N*_e_(*t*) represents the variation in offspring numbers among individuals [9, 10]:

(4)
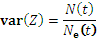

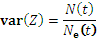


where var(*Z*) is the variation in the number of secondary infections per individual (*R*_0_).

We have previously introduced [7] the phylodynamic transmission parameter (*PTP*) as a value that reflects the transmissions within a population by integrating generation time (*T*) and the variance in the number of secondary infections (var(*Z*)):

(5)
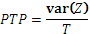

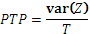


thus, using equations (4) and (5) we get:

(6)
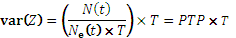

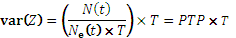


As we have previously shown [7] in a system where a proportion *u* transmits while the rest (*1 - u*) is not transmitting, the distribution of the number of secondary infections *Z* in the whole population (transmitters and non-transmitters; PWID and general population in our case) can be assumed to be approximated by a zero-inflated Poisson distribution such as the mean number of secondary infections per transmitter (*R*_0,a_) (per an injector) is related to the overall mean (expected) number of secondary infections in the population (*R*_0_) by:

(7)
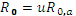

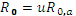


while the variance of secondary infections would be provided by:

(8)
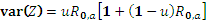

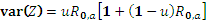


Thus, from equations (6) and (8) we estimate the generation time *T* (the expected time from initial HIV infection to transmission to other individuals) to be

(9)
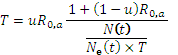

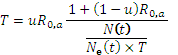


We used the exponential phase of the epidemic growth for calculations of generation time as it expected to be nearly constant over this period. According to both the skyline plot and the estimated HIV prevalent cases curve the exponential phased of the epidemic began around 1995 and ended around 2005 (Fig.1).

All parameters and their explanations are in the Supplementary Table 1.

**Results and interpretations of epidemiological parameters calculations**

We calculated *R*_0,a_ using different values of the duration of infectivity *p* (5-15 years), where 1/*p*=*q* is the overall mortality rate of untreated HIV infected PWID (Suppl. Fig. 2, A). *R*_0,a_ estimates changed from 6 for *p*=5 to 14 for *p*=15. Since HAART was introduced in 1996, treatment coverage of PWID in Russia and Ukraine remained low (only 2% of HIV infected PWID received treatment in early 2000s [11]). Thus, a plausible duration of infectivity for an untreated HIV-infected PWID at that time is unlikely to be more than 8-10 years [12], suggesting that the epidemic in PWID had an *R*_0_ in the range 8-10.

We assumed that the majority of sexual (including both homosexual and heterosexual) transmissions among non-PWID gave rise to a negligibly low number of secondary infections compared to PWID. Heterosexual transmissions were mostly through the sex trade, which is highly correlated with drug use in Russia and Ukraine [13]. It is difficult to quantify the homosexual transmission since homosexuality was highly stigmatized in Russia and Ukraine and was underreported in the surveillance system: in 1990s infections attributed to both homosexual and heterosexual contacts were coded and reported together [14]. We considered *T*<10 days as biologically implausible (viral load is undetectable) [15].

One hypothesis for the explosive growth of the HIV epidemic in Russia and Ukraine is that a particular practice—using blood as a buffer/purifier in the process of domestic drug production—was popular among some PWID [13]. Contamination of large quantities of drugs could facilitate the spread of infection and would allow transmission to multiple recipients generated by a small proportion of PWID. This would translate, within our model framework, into a small proportion of transmitters (those “supplying” blood) driving an epidemic characterised by a very long generation time (around a year, Suppl. Fig.2, B). Translating these dynamics in real-life data means that single individuals should be providing blood for 1 year to purify drugs that were used by 70 PWID. Such a scenario is clinically unlikely due to the large amount of blood that a single individual would need to provide within a long period of time.

Thus we suggest that the most plausible scenario for this HIV epidemic is one characterised by a very short generation time (<1 month), a large proportion of transmitters (70%) and a high number of secondary infections (7 new infections per infected individual). This is compatible with the hypothesis that unsafe injections practiced by a large proportion of PWID (40-90% of Russian/Ukrainian PWID shared instruments or front/backloaded syringes in 1995-2005 [16-18]) may have forced the rapid epidemic spread, which also agrees with our previously reported findings [19]. The fact that a very short generation time was accompanied by a very high value of *R*_0_ is also biologically plausible, since the first month after the infection is the period of the highest infectivity.

**COMPARTMENTAL MODELLING**

**Model description**

We used compartmental Susceptible-Recently Infected-Chronically Infected (Susceptibles – individuals that are not infected, but are at risk of infection; Recently Infected – individuals that acquired infection within the last 6 months; Chronically Infected – individuals that acquired infection longer than 6 months ago) model to describe dynamics of HIV epidemic among PWID in Russia and Ukraine. We used R-project software to estimate epidemiologic parameters given different rates of moving from one compartment to another. Deterministic compartmental modelling has been shown to be effective for epidemiological parameters estimation for epidemics with *R*_0_>1 and large susceptible populations [20], which is the case for our settings. In our model we acknowledge different infectivity during a “recent” (within the first six months after infection) and a “long-term” (after the first six months after HIV acquisition) infectivity periods.

First, we constructed a compartmental model of a natural epidemic without treatment option available (Suppl.Fig.1). This model predicts the number of infected and susceptible individuals over the natural course of the epidemic, for 20 years after HIV introduction into a population of PWID and is described by the following differential equations:


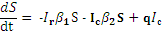

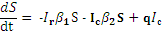


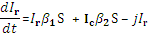

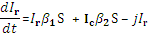


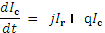

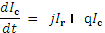


where *S* is the proportion of individuals susceptible to HIV in the PWID population, *I*_r_ is the proportion of recently infected individuals (<6 months since infection), *I*_c_ is the proportion of long-term infected individuals(>6 months since infection).
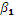

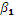
 is a transmission rate coefficient that determines virus transmissibility from individuals recently infected with HIV, whilst
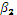

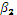
 determines virus transmissibility from individuals who have been infected for at least 6 months; *j* is a rate of progression from the recent to long-term phase of infection (if *c* is the duration of recent phase and *c*=0.5 years, then *j*=1/*c*=1/0.5=2); *q* is a removal rate that indicates overall mortality of HIV infected PWID with untreated infection (if *p* is the duration of infectivity and *p*=10 years, then *q*=1/*p*=1/10=0.1).

We have calculated the *R*(*t*) which is the number of secondary infections per infected individual from the natural epidemic model using next-generation matrix [21]. The eigenvalue *λ* of the matrix FV^-1^ represents the *R*_0_ for the model. In this approach the matrix F represents the rate of appearance of new infections in a compartment, which for our model can be described by the matrix:


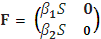

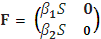
{\displaystyle FV^{-1}}

Similarly, the matrix V represents the rate of transfer of individuals into a compartment by other means (i.e, recently-infected individuals transfer to chronically-infected compartment after 6 months) and is described as:


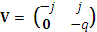


Knowing the F and V allowed us to estimate the reproduction number in our population as:


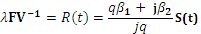


where *S*(*t*) is the change in the compartment of susceptible individuals over time. We have used the value of *R*_0,a_ estimated from the genetic data (10.11 for 10 years of infectivity (*p*, *q*=1/*p*=0.1) and 6 months duration of the recent phase (*c*, *j*=1/*c*=2)) to estimate the transmission rate coefficients (
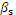

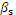
). We have also used the data from another study that showed that transmission hazard during the recent period is 5.3 (95% CI 0.79-57) times higher compared to the long-term infection period (
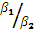

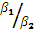
) [22]. This estimate comes from a study of an epidemic driven by sexual transmissions among heterosexual individuals, but was used in our model as there is no similar estimate for PWID transmissions. To estimate the uncertainty of our model we have estimated the credibility intervals for the model compartments using the 95% credibility intervals from the skyline plot estimated from the *env* sequences (Suppl. Fig. 3). We have also conducted sensitivity analysis on the difference in
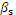

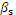
 (Suppl.Fig.4).

To get the estimates of different compartments of the population throughout time we used rates known from the literature. The removal rate *q* is calculated by the infectivity period *p* (assuming the natural history of HIV infection in absence of treatment the time to AIDS/death *p* ~10 years, thus 10 years from the moment of infection to removal, *j*=1/10) and the transition rate *j* is calculated by the duration of recent infection phase *c* ~ 0.5 years, *j*=1/0.5=2)

We have also assumed that the removal rate (infected individual dying from any cause) is equal to the rate at which individuals become susceptible (start drug use in this case); consequently, we assumed that population size is constant throughout time. As the input parameters for the first model, we assumed that the proportion of recently infected individuals in the population at initial time point was 0.0001, consequently, the proportion of susceptibles was 0.9999.

We further advanced this model to include the compartment of treated individuals (highlighted with a red dotted line in Suppl. Fig.1). This model predicts the impact of treatment provided to individuals at different stages of disease (this model was applied to a new epidemic/outbreak and also to an established epidemic) and is described as:


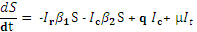

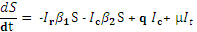


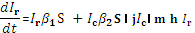

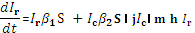


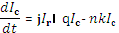

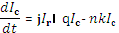


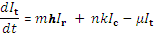

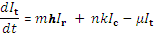


where *S* is the proportion of individuals susceptible to HIV in the population, *I*_r_ is the proportion of recently HIV infected individuals, *I*_c_ is the proportion of long-term infected individuals, *I*_t_ is the proportion of individuals on treatment.
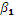

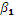
 is a transmission rate coefficient that determines virus transmissibility from individuals recently infected with HIV, whilst
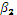

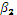
 determines virus transmissibility from individuals who have been infected for more than 6 months; *j* is the rate of progression from recent to long-term phase of infection; *q* is a removal rate that indicates overall mortality of HIV infected PWID with untreated infection; *m* is the rate with which recently infected individuals are engaged into treatment; *n* is the rate with which long-term infected individuals are engaged into treatment; *h* is the proportion of recently infected individuals on treatment; *k* is the proportion of long-term infected individuals on treatment;
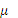

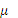
is the rate at which HIV individuals on treatment die.

For the model with treatment compartment
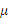

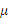
 is calculated assuming that the life expectancy of an HIV-infected person with suppressed viral load would be similar to the general population in Russia and Ukraine in 1990s (70 years,
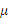

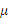
 =1/70). To simplify, we also assume that those individuals that enter a treatment program, stay in treatment, have undetectable viral load and do not infect susceptible individuals. In reality individuals might drop the antiretroviral treatment program or the efficiency of the antiretroviral treatment might be suboptimal. Here we do not explore the effect of treatment drop-outs and virological failures in interventions; thus, the treatment coverage that we are modelling is the ideal scenario. Current HAART regimens achieve virological response in 80-90% of the individuals in treatment in developing countries [23-25], thus we would normally expect higher than the modelled coverage to be required in real-life interventions (i.e, 56-63% instead of 50% in the current model) to observe the dynamics suggested by the model.

We modelled several scenarios for a new epidemic/outbreak (Fig. 2, panels A-D) and for a well-established epidemic (Fig. 2, panels E-H). First, we have compared scenarios when none of the recently infected individuals are on treatment (*h*=0) and 50% of long-term infected individuals receive treatment within the first 4 years after they got infected (*n*=0.25, *k* =0.5) (Fig. 2 A and E) with the scenario when 25% of recently infected individuals and 50% of long-term infected individuals receive treatment (within the first 3 months and the first 4 years, respectively; *h=*0.25, *m=*4.0, *n=*0.25, *k=*0.5) (Fig. 2 B and F). As the initial parameters, for the settings with a new epidemic/outbreak, we assumed that the proportion of recently infected individuals in the population was 0.0001 and the proportion of susceptible was 0.9999. For the scenario of advanced epidemic we assumed that the PWID population consists of 0.01% of recently infected, 10% of individuals on treatment, 20% of long-term infected individuals not on treatment, and the rest 69.99% are susceptible. Then we also introduce a scenario, when the *R*(*t*) is reduced by 60% through the introduction of preventive interventions (Fig. 2 C,D and G,H), thus, reducing the
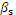

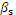
 by 60%.

For all the scenarios we have conducted sensitivity analysis on the proportion of individuals on treatment.

SENSITIVITY AND LIMITATIONS

**Sensitivity analyses**

We conducted sensitivity analyses to test the robustness of our estimates against assumptions on infectivity and the difference in transmission rate coefficients (
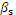

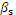
 in Suppl. Fig.1). The infectivity period *p* influenced the estimates significantly changing *R*_0,a_ from around 6 secondary infections per infected individual assuming *p*=5 years to around 14 secondary infections per infected individual for *p*=15 years (Suppl. Fig.2, A). We chose *p*=10 years as the most plausible scenario of natural history of progression towards AIDS for settings with lack of prevention and limited treatment options (as Russia and Ukraine in late 1990s) [12]. Additionally, *p*<5 years resulted in a biologically implausible generation time (10 days or less) given that the proportion of transmitters was more than 70%. For the compartmental modelling, we also tested for sensitivity against assumptions on the infectivity period and it was not sensitive for *p*≥4 years. Furthermore, the sensitivity analysis on the changes of the transmission rate coefficients throughout the infectivity period showed that even if the transmission rate coefficient during the recent phase of infection is only slightly higher or equal to the rate coefficient throughout the remainder of infectivity period, the proportion of new infections attributable to recently infected individuals stabilized at around 5% after 15 years (see Suppl.Fig.4). As expected, the proportion of transmitters changed the estimates of generation time: the bigger the proportion of transmitters – the lower the generation time (Suppl. Fig. 2, B).

Additionally, we have tested a renewal of the PWID population in the compartmental model (Suppl. Fig. 7). More specifically we have modelled a scenario where individuals in antiretroviral treatment quit drug injection with a rate of 1 every 20 person-years. We then add new injectors to the compartment of “Susceptibles” at the same rate. In this scenario, 0% of recently infected individuals and 50% of long-term infected individuals are provided with treatment while no other preventive programs are introduced, similarly to scenario presented in the Fig.2 A. In this case, the dynamics and prevalence stay the same, but the proportion of people in compartments of “Infected” and “Treated” changes. Thus, after 20 years 52% of PWID are expected to be in treatment; 28% of them chronically and 2% recently-infected, not receiving ART; summing up the overall prevalence in the population at 82%, compared to 83% (CI 73-87%) in our original scenario with no drug injection quitting rate.

**Limitations-Assumptions on network effects**

One of the basic assumptions of compartmental modelling is that it assumes panmictic populations – populations with random mixing within compartments, such that individuals have no preferences when choosing partners and establishing connections that can lead to transmissions. However, it is well documented that populations of PWID have network structure and a social network approach might provide a more realistic description of the epidemic dynamics of spread among PWID [26]. In our modelling we assume constant rate at which recent and long-term infected individuals come in contact with susceptible individuals over time. The long-term infected individuals after some time have lower infectiousness, which under a panmictic model reduces the transmission rate as the probability of contact between a susceptible and a highly infectious recently infected is reduced, creating a suboptimal saturation/protection of the epidemic spread. Under some network settings this might however not be true. Some network structures might allow only contact between susceptibles and long-term infected individuals and, thus, prevent susceptible individuals from contact with the highly infectious acutely infected creating a “firewall” effect [27-29].

We believe that this effect is less likely to bias estimates that come from a very large nation-wide epidemic that involves more than 1,000,000 individuals, as higher and/or lower transmission rates would be averaged over a large sample. However, further research is needed that will aim at accounting for the network structure of a population when using this method to describe HIV transmission dynamics in PWID.

**SUPPLEMENTARY FIGURES**

Suppl.Fig.1 **Suppl. Fig.1. Compartmental model that describes natural HIV progression with and without a treatment compartment (the treatment compartment is highlighted with a red dotted line in the figure).** Each box represents compartments within a hypothetical PWID population. *β*_1,_ *β*_2_ – HIV transmission rate coefficients for transmissions from recently and long-term infected individuals, respectively. Other symbols stand for rates with which individuals move from one group (compartment) to another: *j* – rate of progression from recently infected to long-term phase of infection; *q*,
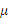
 – removal rates that indicate mortality of HIV infected individuals with untreated and treated HIV infection, respectively; *m*, *n* – rates of involvement in treatment of recently and long-term infected individuals respectively.


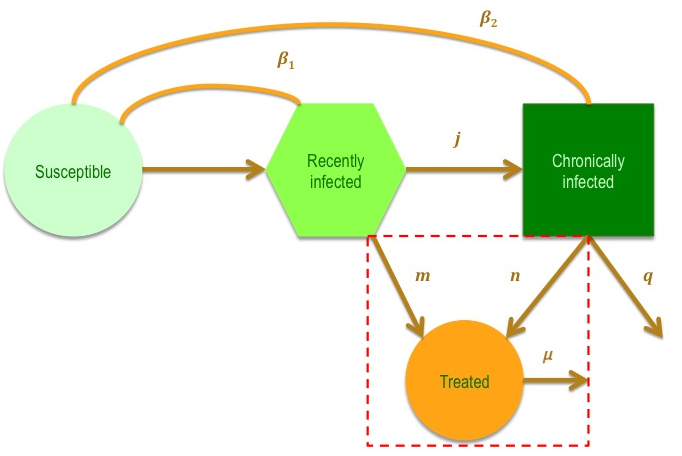


Suppl. Fig.2 **Suppl. Fig.2. Estimates of the epidemiologic parameters.** A) Estimates of the range of *R*_0,a_ values given different duration of infectivity. B) Estimates of the range of generation time values given different proportion of transmitters, assuming the duration of infection to be 10 years and *R*_0,a_ to be 10.1.


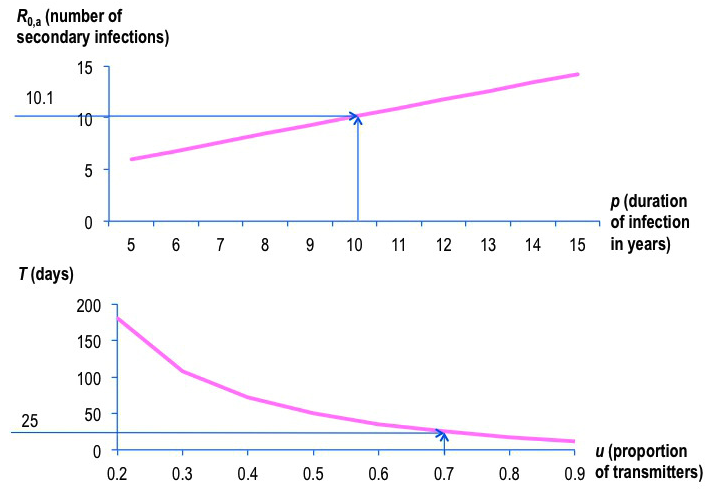


Suppl. Fig. 3 **Suppl. Fig. 3. The results of the natural epidemic model: A) The dynamics in the compartments of Susceptible and Chronically infected; B) The dynamics in the compartment of recently infected.** For the initial parameters, we assumed that the proportion of recently infected individuals in the population was 0.0001 and the proportion of susceptibles was 0.9999. The credibility intervals estimated from the Bayesian skyline plot estimated from the env sequences.


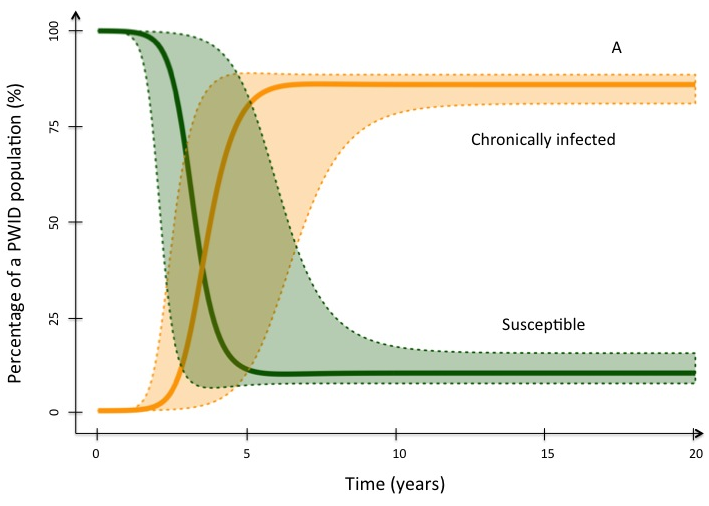


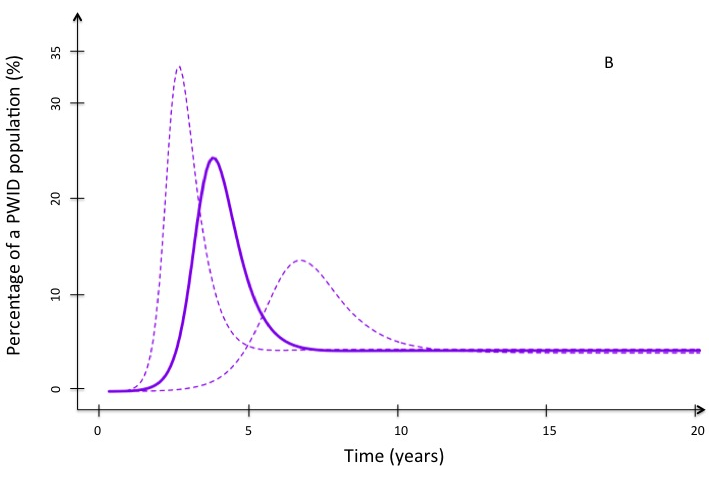


Suppl. Fig.4 **Suppl.Fig.4. Proportion of new HIV infections in the population attributed to recently infected individuals in natural epidemic model.** We assumed different transmission rates: from the same transmission rate during the recent and the long-term phase of the infection to the 6 times higher transmission rate during the recent phase. In our model we assumed that the transmission rates is 5.3 times higher during recent infection.


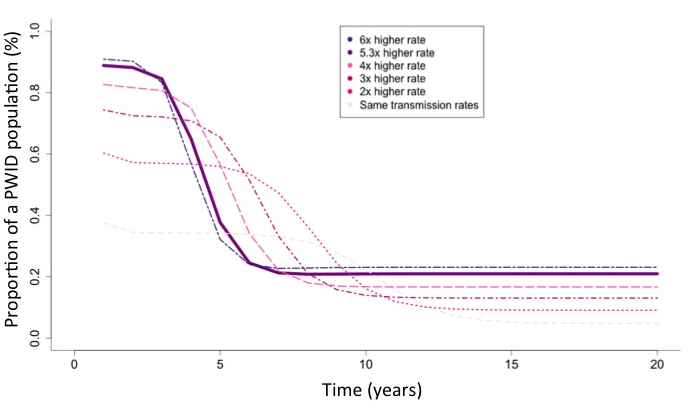


Suppl. Fig.5 **Suppl.Fig.5. The phylogenetic tree constructed using the reference dataset A of pol sequences.**


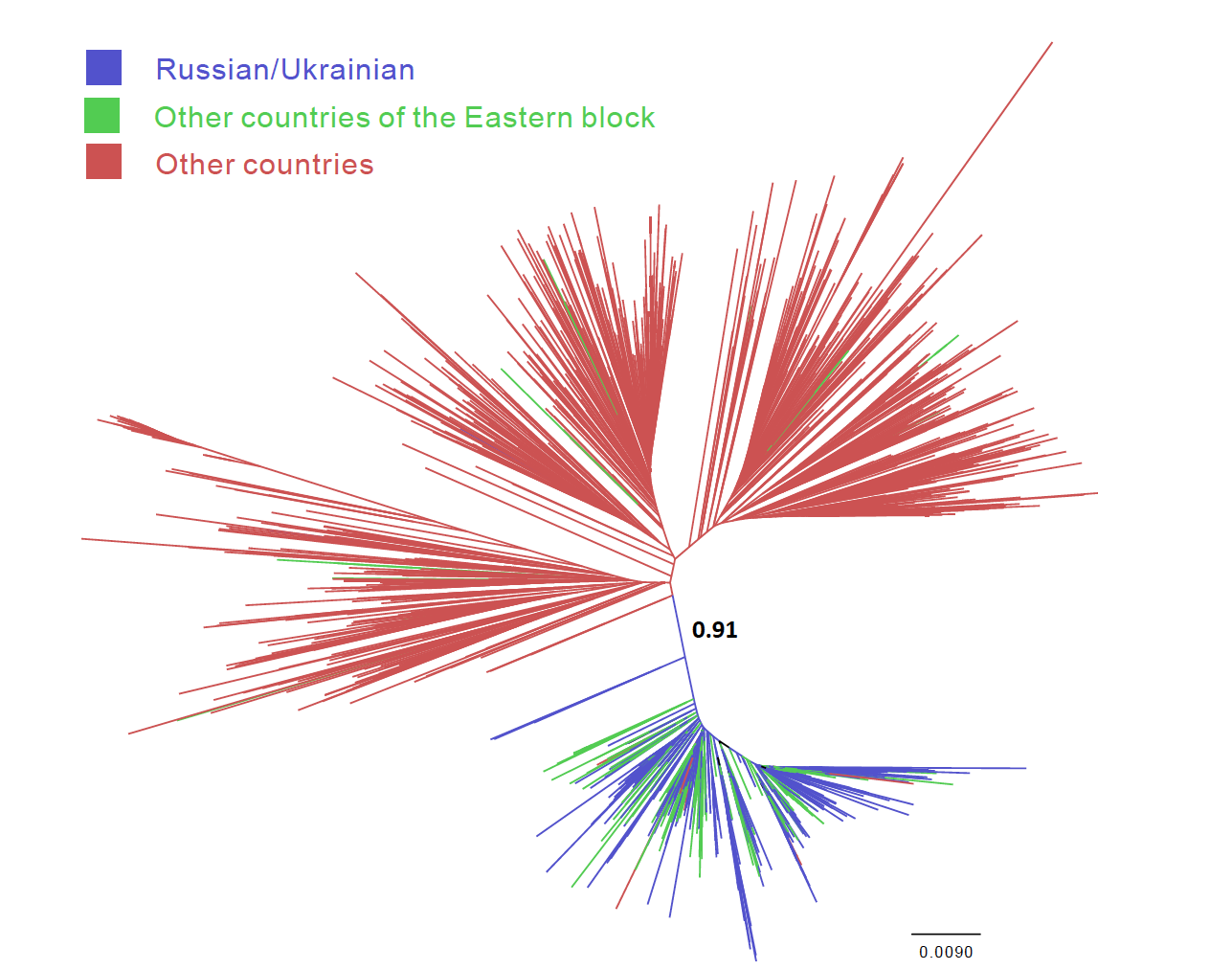


Suppl.Fig.6 **Suppl.Fig.6** Skyline plots reconstructed using partial nucleotide sequences from *pol* (A) and *env* (B) genes. Red lines represent the 95% highest posterior density (HPD) intervals.


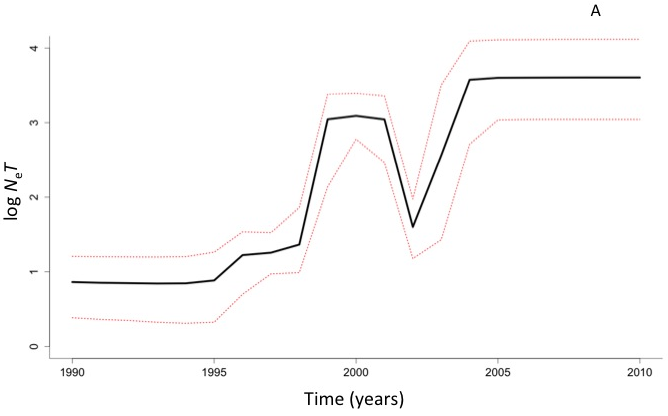


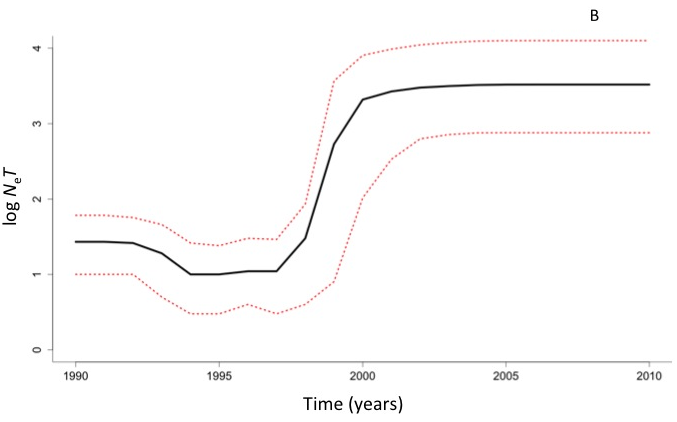


Suppl. Fig. 7 **Suppl.Fig.7***.* **Results of the compartmental model representing HIV spread under a treatment scenario when the PWID population is renewed (quit under antiretroviral and recruit in the Susceptibles).** In this scenario, 0% of recently infected individuals and 50% of long-term infected individuals (similar scenario to Fig.2 A) are provided with treatment while no other preventive programs are introduced. Additionally, the susceptible PWID population is renewed as a result of quitting injection whilst on antiretroviral treatment: a) PWID quit injecting when introduced and sustained in antiretroviral treatment with a rate of 1 PWID quitting every 20 person-years of treatment, b) susceptible injectors are recruited with the same rate.


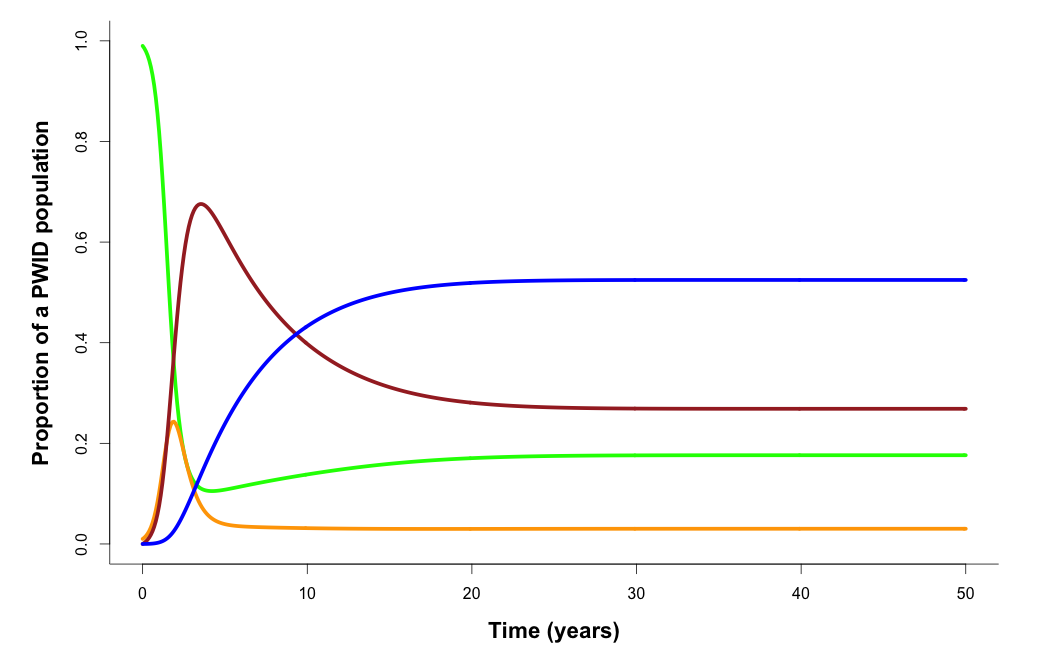


**REFERENCES**

1. Tamura K, Stecher G, Peterson D, Filipski A, Kumar S. MEGA6: Molecular Evolutionary Genetics Analysis version 6.0. *Mol Biol Evol* 2013,**30**:2725-2729.

2. Saad MD, Shcherbinskaya AM, Nadai Y, Kruglov YV, Antonenko SV, Lyullchuk MG*, et al.* Molecular epidemiology of HIV Type 1 in Ukraine: birthplace of an epidemic. *AIDS Res Hum Retroviruses* 2006,**22**:709-714.

3. Nabatov AA, Kravchenko ON, Lyulchuk MG, Shcherbinskaya AM, Lukashov VV. Simultaneous introduction of HIV type 1 subtype A and B viruses into injecting drug users in southern Ukraine at the beginning of the epidemic in the former Soviet Union. *AIDS Res Hum Retroviruses* 2002,**18**:891-895.

4. Drummond AJ, Rambaut A. BEAST: Bayesian evolutionary analysis by sampling trees. *BMC Evol Biol* 2007,**7**:214.

5. Drummond AJ, Ho SY, Phillips MJ, Rambaut A. Relaxed phylogenetics and dating with confidence. *PLoS Biol* 2006,**4**:e88.

6. HIV/AIDS. JUNPo. UNAIDS: Indicator registry. In. Geneva, Switzerland: Joint United Nations Programme on HIV/AIDS; 2015.

7. Magiorkinis G, Sypsa V, Magiorkinis E, Paraskevis D, Katsoulidou A, Belshaw R*, et al.* Integrating phylodynamics and epidemiology to estimate transmission diversity in viral epidemics. *PLoS Comput Biol* 2013,**9**:e1002876.

8. Keeling Mj RP. *Modeling Infectious Diseases in Humans and*

*Animals*. Princeton, New Jersey: Princeton University Press; 2008.

9. Tavare S, Balding DJ, Griffiths RC, Donnelly P. Inferring coalescence times from DNA sequence data. *Genetics* 1997,**145**:505-518.

10. Kingman J. On the genealogy of large populations. *J Appl Probability* 1982,**19**:27-43.

11. Mathers BM, Degenhardt L, Ali H, Wiessing L, Hickman M, Mattick RP*, et al.* HIV prevention, treatment, and care services for people who inject drugs: a systematic review of global, regional, and national coverage. *Lancet* 2010,**375**:1014-1028.

12. Longini IM, Jr., Clark WS, Byers RH, Ward JW, Darrow WW, Lemp GF*, et al.* Statistical analysis of the stages of HIV infection using a Markov model. *Stat Med* 1989,**8**:831-843.

13. Artyuh O.R. BOM, Bochkova L.V., Galych Y.P., Halustyan Y.M., Dikova-Favorska D. M., Zlobina O.G., Levchuk N.M., Lyutyi V.P., Marcynovska V.A., Mykytyuk T.P., Morozov V.F., Petrovskyi O.M., Shamota T.S., Yaremenko O.O. Analytical report. Monitoring of behavior of injecting drug users as a component of second generation epidemiological surveillance. In. Kyiv: International HIV/AIDS Alliance in Ukraine; 2005.

14. Beyrer C, Wirtz AL, Walker D, Johns B, Sifakis F, Baral SD. The Global HIV Epidemics among Men Who Have Sex with Men Introduction. *Global Hiv Epidemics among Men Who Have Sex with Men* 2011:1-4.

15. McMichael AJ, Borrow P, Tomaras GD, Goonetilleke N, Haynes BF. The immune response during acute HIV-1 infection: clues for vaccine development. *Nat Rev Immunol* 2010,**10**:11-23.

16. Booth RE, Lehman WE, Dvoryak S, Brewster JT, Sinitsyna L. Interventions with injection drug users in Ukraine. *Addiction* 2009,**104**:1864-1873.

17. Grund J-PC. *A Candle Lit from Both Sides: The Epidemic of HIV Infection in Central and Eastern Europe*. Westport, Ct. USA: Greenwood Press; 2001.

18. Rhodes T, Lowndes C, Judd A, Mikhailova LA, Sarang A, Rylkov A*, et al.* Explosive spread and high prevalence of HIV infection among injecting drug users in Togliatti City, Russia. *AIDS* 2002,**16**:F25-31.

19. Magiorkinis G. PD, Pybus OG., Karamitros T., Vasylyeva T., Bobkova M., Hatzakis M. HIV-1 epidemic in Russia: an evolutionary epidemiology

analysis. In: *Lancet*. Online; 2014.

20. Popinga A, Vaughan T, Stadler T, Drummond AJ. Inferring epidemiological dynamics with Bayesian coalescent inference: the merits of deterministic and stochastic models. *Genetics* 2015,**199**:595-607.

21. Diekmann O, Heesterbeek JA, Roberts MG. The construction of next-generation matrices for compartmental epidemic models. *J R Soc Interface* 2010,**7**:873-885.

22. Bellan SE, Dushoff J, Galvani AP, Meyers LA. Reassessment of HIV-1 Acute Phase Infectivity: Accounting for Heterogeneity and Study Design with Simulated Cohorts. *PLoS Med* 2015,**12**:e1001801.

23. Mosha F, Ledwaba J, Ndugulile F, Ng'ang'a Z, Nsubuga P, Morris L*, et al.* Clinical and virological response to antiretroviral drugs among HIV patients on first-line treatment in Dar-es-Salaam, Tanzania. *J Infect Dev Ctries* 2014,**8**:845-852.

24. Ugbena R, Aberle-Grasse J, Diallo K, Bassey O, Jelpe T, Rottinghaus E*, et al.* Virological response and HIV drug resistance 12 months after antiretroviral therapy initiation at 2 clinics in Nigeria. *Clin Infect Dis* 2012,**54 Suppl 4**:S375-380.

25. Abdissa A, Yilma D, Fonager J, Audelin AM, Christensen LH, Olsen MF*, et al.* Drug resistance in HIV patients with virological failure or slow virological response to antiretroviral therapy in Ethiopia. *BMC Infect Dis* 2014,**14**:181.

26. Friedman SR, Neaigus A, Jose B, Curtis R, Goldstein M, Ildefonso G*, et al.* Sociometric risk networks and risk for HIV infection. *Am J Public Health* 1997,**87**:1289-1296.

27. Friedman SR, Kottiri BJ, Neaigus A, Curtis R, Vermund SH, Des Jarlais DC. Network-related mechanisms may help explain long-term HIV-1 seroprevalence levels that remain high but do not approach population-group saturation. *Am J Epidemiol* 2000,**152**:913-922.

28. Khan B, Dombrowski K, Saad M, McLean K, Friedman S. Network Firewall Dynamics and the Subsaturation Stabilization of HIV. *Discrete Dyn Nat Soc* 2013,**2013**:720818.

29. Dombrowski K, Curtis R, Friedman S, Khan B. Topological and Historical Considerations for Infectious Disease Transmission among Injecting Drug Users in Bushwick, Brooklyn (USA). *World J AIDS* 2013,**3**:1-9.

**Supplementary Table 1**

| Parameter | Explanation | Page; MT - main text,  ST - supplementary text |
| --- | --- | --- |
| *c* | Duration of recent phase | ST 6 |
| F | Matrix that represents the rate of appearance of new infections in a compartment in the model | ST 6 |
| *h* | Proportion of recently infected individuals on treatment | ST 7 |
| *I*_c_ | Proportion of long-term infected individuals (>6 months since infection) | ST 6 |
| *I*_r_ | Proportion of recently infected individuals (<6 months since infection) | ST 6 |
| *I*_t_ | Proportion of individuals on treatment | ST 7 |
| *j* | Rate of progression from the recent to long-term phase of infection | MT 5, ST 3 |
| *k* | Proportion of long-term infected individuals on treatment | ST 7 |
| *m* | Rate with which recently infected individuals are engaged into treatment | ST 7 |
| *n* | Rate with which long-term infected individuals are engaged into treatment | ST 7 |
| *N(t)* | Estimate of HIV prevalence at time *t* | ST 4 |
| *N*_e_(0) | Effective population size at the baseline of the exponential growth phase | MT 5, ST 3 |
| *N*_e_(*t*) | Effective population size at time *t* | MT 5, ST 3 |
| *p* | Duration of infectivity | MT 7, ST 5 |
| *PTP* | Phylodynamic transmission parameter | ST 4 |
| *q* | Overall mortality rate of HIV infected individuals | MT 5, ST 3 |
| *R*_0_ | Number of secondary infections | MT 5, ST 3 |
| *R*_0,a_ | Mean number of secondary infections per transmitter (per an injector) | MT 5, ST 4 |
| *S* | Proportion of individuals susceptible to HIV in a PWID population | ST 6 |
| *T* | Generation time | MT 6, ST 3 |
| *u* | Proportion of transmitters in HIV-infected population | MT 5, ST 4 |
| V | Matrix that represents the rate of transfer of individuals into a compartment by other means | ST 6 |
| var (*Z*) | Variation in the number of secondary infections per individual | ST 4 |
| 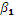 | Transmission rate coefficient that determines virus transmissibility from individuals recently infected with HIV | ST 6 |
| 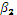 | Transmission rate coefficient that determines virus transmissibility from individuals who have been infected for at least 6 months | ST 6 |
| *λ* | Eigenvalue of a matrix | ST 6 |
| *μ* | Rate at which HIV individuals on treatment die | ST 7 |
| *ω* | Average removal rate in a population | ST 3 |

**Supplementary Table 2**

**Dataset A**

| 97CDKTB48 |
| --- |
| FJ822055 |
| FJ822064 |
| FJ822065 |
| EU345595 |
| EU345596 |
| FJ822066 |
| FJ822067 |
| FJ822068 |
| FJ822069 |
| FJ822070 |
| FJ822071 |
| FJ822072 |
| FJ822073 |
| FJ822056 |
| FJ822074 |
| FJ822076 |
| FJ822077 |
| FJ822078 |
| FJ822080 |
| FJ822082 |
| FJ822083 |
| FJ822057 |
| FJ822084 |
| FJ822085 |
| FJ822086 |
| FJ822087 |
| FJ822090 |
| FJ822091 |
| FJ822092 |
| FJ822093 |
| FJ822058 |
| FJ822094 |
| FJ822095 |
| FJ822096 |
| FJ822097 |
| FJ822098 |
| FJ822100 |
| FJ822101 |
| FJ822102 |
| FJ822059 |
| FJ822104 |
| FJ822105 |
| FJ822106 |
| FJ822107 |
| FJ822108 |
| FJ822109 |
| EU345598 |
| FJ822110 |
| FJ822111 |
| FJ822113 |
| FJ822060 |
| FJ822115 |
| FJ822116 |
| FJ822117 |
| FJ822118 |
| FJ822119 |
| FJ822120 |
| FJ822121 |
| FJ822122 |
| FJ822123 |
| FJ822061 |
| FJ822124 |
| FJ822125 |
| FJ822126 |
| FJ822127 |
| FJ822129 |
| FJ822130 |
| FJ822131 |
| FJ822132 |
| FJ822133 |
| FJ822062 |
| FJ822134 |
| EU345599 |
| EU345600 |
| EU345601 |
| FJ822063 |
| EU345602 |
| EU345603 |
| EU345604 |
| EU345606 |
| EU345841 |
| EU345842 |
| EU345844 |
| GQ867609 |
| GQ867624 |
| GQ867605 |
| GQ867628 |
| GQ867630 |
| GQ867635 |
| GQ867618 |
| GQ867616 |
| GQ867632 |
| GQ867633 |
| GQ867620 |
| GQ867627 |
| GQ867608 |
| GQ867629 |
| GQ867631 |
| GQ867606 |
| GQ867607 |
| GQ867614 |
| GQ867612 |
| GQ867604 |
| GQ867636 |
| GQ867617 |
| GQ867626 |
| GQ867619 |
| GQ867615 |
| GQ867625 |
| GQ867623 |
| GQ867621 |
| GQ867597 |
| GQ867610 |
| GQ867611 |
| GQ867599 |
| GQ867634 |
| GQ867622 |
| GQ867603 |
| GQ867602 |
| GQ867601 |
| GQ867598 |
| GQ867600 |
| EU345614 |
| EU345615 |
| EU345616 |
| EU345617 |
| EU345618 |
| EU345619 |
| EU345620 |
| EU345621 |
| EU345622 |
| EU345623 |
| EU345624 |
| EU345625 |
| EU345626 |
| EU345627 |
| EU345628 |
| EU345629 |
| EU345630 |
| EU345631 |
| EU345632 |
| EU345633 |
| EU345634 |
| EU345635 |
| EU345636 |
| EU345637 |
| EU345638 |
| EU345639 |
| EU345640 |
| EU345641 |
| EU345642 |
| EU345643 |
| EU345644 |
| EU345645 |
| EU345646 |
| EU345647 |
| EU345648 |
| EU345649 |
| EU345650 |
| EU345651 |
| EU345652 |
| EU345653 |
| EU345654 |
| EU345655 |
| EU345656 |
| EU345657 |
| EU345658 |
| EU345659 |
| EU345660 |
| EU345661 |
| EU345662 |
| EU345663 |
| EU345664 |
| EU345665 |
| EU345666 |
| EU345667 |
| EU345668 |
| EU345669 |
| EU345670 |
| EU345671 |
| EU345673 |
| EU345674 |
| EU345675 |
| EU345676 |
| EU345677 |
| EU345678 |
| EU345679 |
| EU345680 |
| EU345681 |
| EU345682 |
| EU345683 |
| EU345684 |
| EU345685 |
| EU345686 |
| EU345687 |
| EU345688 |
| EU345689 |
| EU345690 |
| EU345691 |
| EU345692 |
| EU345693 |
| EU345694 |
| EU345700 |
| EU345701 |
| EU345847 |
| EU345702 |
| EU345703 |
| EU345704 |
| EU345705 |
| EU345706 |
| EU345848 |
| EU345707 |
| EU345708 |
| EU345709 |
| EU345711 |
| EU345713 |
| EU345714 |
| EU345715 |
| EU345716 |
| EU345718 |
| EU345719 |
| EU345721 |
| EU345722 |
| EU345852 |
| EU345723 |
| EU345725 |
| EU345726 |
| EU345727 |
| EU345728 |
| EU345729 |
| EU345730 |
| EU345731 |
| EU345732 |
| EU345733 |
| EU345734 |
| EU345735 |
| EU345736 |
| EU345737 |
| EU345738 |
| EU345739 |
| EU345740 |
| EU345741 |
| EU345743 |
| EU345744 |
| EU345745 |
| EU345746 |
| EU345747 |
| EU345748 |
| EU345749 |
| EU345750 |
| EU345751 |
| EU345752 |
| EU345753 |
| EU345754 |
| EU345755 |
| EU345756 |
| EU345758 |
| EU345759 |
| EU345760 |
| EU345761 |
| EU345762 |
| EU345763 |
| EU345764 |
| EU345765 |
| EU345766 |
| EU345767 |
| EU345770 |
| EU345771 |
| EU345773 |
| FJ009713 |
| FJ009714 |
| FJ009715 |
| FJ009716 |
| FJ009717 |
| FJ009718 |
| FJ009719 |
| FJ009720 |
| FJ009721 |
| FJ009722 |
| FJ009723 |
| FJ009724 |
| FJ009725 |
| FJ009726 |
| FJ009727 |
| FJ009728 |
| FJ009729 |
| FJ009730 |
| FJ009731 |
| FJ009732 |
| FJ009733 |
| FJ009734 |
| FJ009735 |
| FJ009736 |
| FJ009737 |
| FJ009738 |
| FJ009739 |
| FJ009740 |
| FJ009741 |
| FJ009742 |
| FJ009743 |
| FJ009744 |
| FJ009745 |
| FJ009746 |
| FJ009747 |
| FJ009748 |
| FJ009749 |
| FJ009750 |
| FJ009751 |
| FJ009752 |
| FJ009753 |
| FJ009754 |
| FJ009755 |
| FJ009756 |
| FJ009757 |
| FJ009759 |
| FJ009760 |
| FJ009762 |
| FJ009763 |
| FJ009764 |
| FJ009765 |
| FJ009766 |
| FJ009767 |
| FJ009768 |
| FJ009770 |
| FJ009772 |
| FJ009773 |
| FJ009758 |
| EU345780 |
| EU345781 |
| EU345784 |
| EU345785 |
| EU345854 |
| EU345786 |
| EU345855 |
| EU345787 |
| EU345858 |
| EU345788 |
| EU345859 |
| EU345789 |
| FJ009775 |
| FJ009777 |
| FJ009778 |
| EU345791 |
| EU345866 |
| EU345867 |
| EU345868 |
| EU345869 |
| EU345870 |
| EU345792 |
| EU345871 |
| EU345872 |
| EU345873 |
| EU345874 |
| EU345875 |
| EU345876 |
| EU345794 |
| EU345877 |
| EU345878 |
| EU345879 |
| EU345880 |
| EU345795 |
| EU345881 |
| EU345882 |
| EU345796 |
| EU345883 |
| EU345797 |
| EU345884 |
| EU345885 |
| EU345886 |
| EU345887 |
| EU345888 |
| EU345889 |
| EU345890 |
| EU345891 |
| EU345892 |
| EU345893 |
| EU345894 |
| EU345799 |
| EU345800 |
| EU345895 |
| EU345896 |
| EU345801 |
| EU345897 |
| EU345898 |
| EU345899 |
| EU345802 |
| EU345900 |
| EU345901 |
| EU345803 |
| EU345902 |
| EU345903 |
| EU345904 |
| EU345905 |
| EU345921 |
| EU345806 |
| EU345924 |
| EU345925 |
| EU345926 |
| EF545110 |
| EU345936 |
| EU345940 |
| EU345943 |
| FJ009783 |
| FJ009784 |
| FJ009786 |
| FJ009789 |
| FJ009790 |
| FJ009791 |
| FJ009792 |
| FJ009793 |
| FJ009794 |
| FJ009795 |
| FJ009796 |
| FJ009797 |
| FJ009798 |
| FJ009799 |
| FJ009800 |
| FJ009801 |
| FJ009802 |
| FJ009803 |
| FJ009804 |
| FJ009805 |
| FJ009806 |
| FJ009807 |
| FJ009808 |
| FJ009809 |
| FJ009811 |
| FJ009812 |
| FJ009813 |
| EU345807 |
| EU345808 |
| EU345809 |
| EU345810 |
| EU345811 |
| EU345812 |
| EU345813 |
| EU345814 |
| EU345815 |
| EU345816 |
| EU345817 |
| EU345818 |
| EU345819 |
| EU345820 |
| EU345821 |
| EU345822 |
| EU345823 |
| EU345824 |
| EU345825 |
| EU345826 |
| EU345827 |
| EU345828 |
| EU345829 |
| EU345830 |
| SP811s2004 |
| 00051s2000 |
| SP812s2004 |
| 02KZYUZ300408 |
| 02KZYUZ300412 |
| 02KZYUZ300413 |
| 02KZYUZ300414 |
| 02KZYUZ300415 |
| 02KZYUZ300418 |
| 02KZYUZ300420 |
| 02KZYUZ300421 |
| 02KZYUZ300425 |
| 02KZYUZ300426 |
| 02KZKAR300435 |
| 02KZKAR300438 |
| 02KZKAR300439 |
| 02KZKAR300441 |
| 02KZKAR300442 |
| 02KZKAR300443 |
| 02KZKAR300444 |
| 02KZKAR300445 |
| 02KZKAR300446 |
| 02KZKAR300449 |
| 02KZKAR300451 |
| 02KZKAR300453 |
| 02KZKAR300454 |
| 02KZKAR300455 |
| 02KZKAR300457 |
| 02KZKAR300459 |
| 02KZKAR300460 |
| 02KZKAR300462 |
| 02KZKAR300464 |
| 02KZKAR300465 |
| 02KZKAR300466 |
| 02KZKAR300467 |
| 02KZPAV300480 |
| 02KZPAV300484 |
| 02KZPAV300487 |
| 02KZPAV300493 |
| 02KZPAV300497 |
| 02KZPAV300502 |
| 03KZALM300210 |
| 03KZ300215 |
| 03KZALM300216 |
| 03KZALM300219 |
| 03KZALM300220 |
| 03KZALM300230 |
| 03KZMAN300232 |
| 03KZ300235 |
| 03KZ300243 |
| 03KZVOS300245 |
| 03KZVOS300246 |
| 03KZVOS300248 |
| 03KZVOS300249 |
| 03KZVOS300254 |
| 03KZVOS300255 |
| 03KZVOS300256 |
| 03KZVOS300259 |
| 03KZVOS300260 |
| 03KZVOS300261 |
| 03KZVOS300262 |
| 03KZVOS300263 |
| 03KZVOS300264 |
| 03KZVOS300265 |
| 03KZVOS300267 |
| 03KZYUZ300273 |
| 03KZYUZ300278 |
| 03KZYUZ300279 |
| 03KZYUZ300280 |
| 03KZYUZ300281 |
| 03KZYUZ300282 |
| 03KZYUZ300283 |
| 03KZYUZ300284 |
| 03KZYUZ300285 |
| 03KZYUZ300287 |
| 03KZYUZ300288 |
| 03KZYUZ300290 |
| 03KZYUZ300291 |
| 03KZYUZ300292 |
| 03KZYUZ300293 |
| 03KZYUZ300296 |
| 03KZYUZ300300 |
| 03KZYUZ300302 |
| 01UADN110 |
| 01UADN113 |
| 01UADN114 |
| 01UADN116 |
| 01UADN119 |
| 01UADN121 |
| 01UADN127 |
| 01UADN128 |
| 01UADN129 |
| 01UADN136 |
| 01UADN137 |
| 01UADN139 |
| 01UADN141 |
| 01UADN145 |
| 01UADN147 |
| 01UADN149 |
| 01UADN180 |
| 01UADN216 |
| 01UADN220 |
| 01UADN222 |
| 01UADN224 |
| 01UADN228 |
| 01UAKV099 |
| 01UAKV108 |
| 01UAKV172 |
| 01UAKV178 |
| 01UAKV248 |
| 01UAKV254 |
| 01UAKV269 |
| 01UAOD008 |
| 01UAOD010 |
| 01UAOD025 |
| 01UAOD027 |
| 01UAOD028 |
| 01UAOD035 |
| 01UAOD040 |
| 01UAOD041 |
| 01UAOD049 |
| 01UAOD051 |
| 01UAOD069 |
| 01UAOD070 |
| 01UAOD072 |
| 01UAOD089 |
| 01UAOD095 |
| 01UAOD096 |
| 01UAOD098 |
| 01UAOD102 |
| 01UAOD103 |
| 01UAPol279 |
| 01UAPol286 |
| 01UAPol288 |
| 01UAPol289 |
| 01UAPol290 |
| 01UAPol291 |
| 01UAPol293 |
| 01UAPol295 |
| 01UAPol296 |
| 01UAPol297 |
| 01UAPol298 |
| 01UAPol299 |
| 01UAPol300 |
| 01UAPol302 |
| 01UAPol303 |
| 01UAPol304 |
| 01UAPol310 |
| 01UAPol311 |
| 01UAPol313 |
| 01UAPol314 |
| 02UACR001 |
| 02UACR002 |
| 02UACR003 |
| 02UACR004 |
| 02UACR005 |
| 02UACR006 |
| 02UACR007 |
| 02UACR008 |
| 02UACR009 |
| 02UACR010 |
| 02UACR011 |
| 02UACR012 |
| 02UACR014 |
| 02UACR015 |
| 02UACR016 |
| 02UACR018 |
| 02UACR020 |
| 02UACR021 |
| 02UAKV005 |
| 02UAKV107 |
| 02UAKV114 |
| 02UAKV115 |
| 02UAKV117 |
| 02UAKV121 |
| 02UAKV131 |
| 02UAKV145 |
| 02UAKV149 |
| 02UAKV150 |
| 02UANK022 |
| 02UAKV178 |
| 02UAKV187 |
| 02UAKV180 |
| 02UAKV179 |
| 02UAKV176 |
| 02UAKV156 |
| 02UAKV152 |
| 02UAKV175 |
| 02UAKV182 |
| 01UAOD10 |
| 01UAOD35 |
| 01UAOD89 |
| 02UZ816 |
| 02UZ817 |
| 02UZ818 |
| 02UZ824 |
| 02UZ825 |
| 02UZ827 |
| 02UZ831 |
| 02UZ833 |
| 02UZ834 |
| 02UZ835 |
| 02UZ837 |
| 02UZ838 |
| 02UZ840 |
| 02UZ841 |
| 02UZ842 |
| 02UZ843 |
| 02UZ844 |
| 02UZ847 |
| 02UZ848 |
| 03UZ001 |
| 03UZ002 |
| 03UZ003 |
| 03UZ004 |
| 03UZ005 |
| 03UZ006 |
| 03UZ007 |
| 03UZ010 |
| 03UZ012 |
| 03UZ013 |
| 03UZ015 |
| 03UZ016 |
| 03UZ018 |
| 03UZ019 |
| 03UZ020 |
| 03UZ021 |
| 03UZ022 |
| 03UZ023 |
| 03UZ029 |
| 03UZ030 |
| 03UZ033 |
| 03UZ034 |
| 03UZ036 |
| 03UZ038 |
| 03UZ039 |
| 03UZ041 |
| 03UZ043 |
| 03UZ044 |
| 03UZ046 |
| 03UZ047 |
| 03UZ049 |
| 03UZ051 |
| 03UZ057 |
| 03UZ068 |
| 03UZ069 |
| 03UZ072 |
| 03UZ073 |
| 03UZ076 |
| 02UZ652 |
| 02UZ654 |
| 02UZ659 |
| 02UZ660 |
| 02UZ661 |
| 02UZ663 |
| 02UZ664 |
| 02UZ665 |
| 02UZ667 |
| 02UZ668 |
| 02UZ669 |
| 02UZ670 |
| 02UZ671 |
| 02UZ672 |
| 02UZ676 |
| 02UZ679 |
| 02UZ680 |
| 02UZ687 |
| 02UZ691 |
| 02UZ693 |
| 02UZ695 |
| 02UZ696 |
| 02UZ698 |
| 02UZ702 |
| 02UZ703 |
| 02UZ707 |
| 02UZ771 |
| 02UZ708 |
| 02UZ713 |
| 02UZ723 |
| 02UZ724 |
| 02UZ725 |
| 02UZ726 |
| 02UZ727 |
| 02UZ729 |
| 02UZ730 |
| 02UZ732 |
| 02UZ733 |
| 02UZ734 |
| 02UZ737 |
| 02UZ740 |
| 02UZ741 |
| 02UZ742 |
| 02UZ744 |
| 02UZ745 |
| 02UZ747 |
| 02UZ750 |
| 02UZ752 |
| 02UZ754 |
| 02UZ755 |
| 02UZ756 |
| 02UZ757 |
| 02UZ761 |
| 02UZ762 |
| 02UZ773 |
| 02UZ774 |
| 02UZ775 |
| 02UZ776 |
| 02UZ778 |
| 02UZ784 |
| 02UZ801 |
| 02UZ803 |
| 02UZ807 |
| 02UZ814 |
| GQ477451 |
| GQ477450 |
| GQ477449 |
| GQ477448 |
| GQ477447 |
| GQ477446 |
| GQ477445 |
| GQ477444 |
| GQ477443 |
| GQ477442 |
| GQ477441 |
| EF158043 |
| EF158042 |
| EF158041 |
| EF158040 |
| 01AZ001 |
| 01AZ003 |
| 01AZ004 |
| 01AZ006 |
| 01AZ009 |
| 01AZ014 |
| 01AZ025 |
| 01AZ026 |
| 01AZ030 |
| 01AZ031 |
| 01AZ036 |
| 01AZ038 |
| 02AZ005 |
| 02AZ007 |
| 02AZ015 |
| 02AZ018 |
| 02AZ020 |
| 02AZ023 |
| 02AZ024 |
| 02AZ025 |
| 02AZ028 |
| 02AZ034 |
| 02AZ037 |
| 02AZ038 |
| 02AZ050 |
| 02AZ054 |
| 02AZ058 |
| 02AZ060 |
| 02AZ062 |
| 02AZ066 |
| 02AZ069 |
| 02AZ077 |
| 02AZ080 |
| 02AZ083 |
| 02AZ089 |
| 02AZ093 |
| 02AZ098 |
| 98GEMZ005 |
| 98GEMZ007 |
| 98GEMZ008 |
| 99GEMZ011 |
| 99GEMZ011(2) |
| 99GEMZ012 |
| 99GEMZ013 |
| 03GEMZ002 |
| 03GEMZ003 |
| 03GEMZ007 |
| 03GEMZ008 |
| 03GEMZ009 |
| 03GEMZ011 |
| 03GEMZ012 |
| 03GEMZ014 |
| 03GEMZ016 |
| 03GEMZ017 |
| 03GEMZ020 |
| 03GEMZ021 |
| 03GEMZ022 |
| 03GEMZ024 |
| 03GEMZ027 |
| 03GEMZ030 |
| 03GEMZ031 |
| 03GEMZ032 |
| 03GEMZ034 |
| 03GEMZ035 |
| 03GEMZ036 |
| 03GEMZ038 |
| 03GEMZ040 |
| GU945074 |
| GU945075 |
| GU945077 |
| GU945079 |
| GU945080 |
| GU945082 |
| GU945090 |
| CB_155 |
| CB_156 |
| CB_599 |
| GU945091 |
| GU945092 |
| GU945096 |
| GU945101 |
| GU945102 |
| GU945106 |
| GU945107 |
| GU945108 |
| GU945109 |
| GU945113 |
| GU945127 |
| GU945132 |
| GU945140 |
| GU945141 |
| GU945142 |
| GU945143 |
| GU945160 |
| GU945161 |
| GU945162 |
| GU945163 |
| GU945164 |
| GU945165 |
| GU945166 |
| GU945169 |
| GU945170 |
| GU945173 |
| GU945174 |
| GU945175 |
| GU945189 |
| GU945190 |
| GU945194 |
| 44 |
| UG273 |
| 65 |
| 164 |
| 180 |
| 401 |
| 419 |
| 429 |
| 440 |
| 441 |
| 453 |
| 496 |
| 503 |
| 542 |
| 574 |
| 584 |
| 599 |
| 604 |
| 608 |
| 622 |
| 623 |
| 646 |
| 649 |
| 652 |
| 653 |
| 660 |
| 687 |
| 702 |
| 704 |
| 718NT6W |
| 734 |
| 742 |
| 792 |
| 807 |
| 841 |
| 13 |
| 173 |
| 185 |
| 444 |
| 879 |
| 522 |
| 532 |
| 534 |
| 545 |
| 577 |
| 592 |
| 699 |
| 710 |
| 790 |
| 847 |
| 871 |
| UG275 |
| 96CM_MP512 |
| 97CM_MP582 |
| 97CM_MP640 |
| 98CM_MP1019 |
| 98CM_MP1094 |
| 98CM_MP1173 |
| 00GAB_LBV02_08 |
| 00GAB_LBV18_08 |
| 00GAB_01B |
| 00GAB_03 |
| 00GAB_04B |
| 00GAB_14M |
| 00GAB_18N |
| 00SN_69HPD_4_9_00 |
| 97CD_KCC2 |
| 97CD_KTB13 |
| 02CD_KTB035 |
| 02CD_KS035 |
| 02CD_LBS024 |
| 02CD_KSS013 |
| 02CD_KIST061 |
| 02BU_U1808 |
| 02BU_U2226 |
| 02BU_U2929 |
| 02BU_U3121 |
| 02BU_U0706 |
| 02BU_U1524 |
| 02BU_U1617 |
| 02BU_U1803 |
| 97CM_MP812 |
| 99CM_MP1370 |
| 99CM_MP1433 |
| 99CM_MP1411 |
| DDI579 |
| DDJ360 |
| DDJ369 |
| 02_1354KP |
| 03_9073NR |
| 01_300NG |
| 03_9146NM |
| 01_1378MJ |
| 03_9410NS |
| 03_9366WG |
| 03_9412NS |
| 03_9538NG |
| 03_9456KM |
| 999_7NM |
| 04_0567NR |
| TC025104 |
| cm1657 |
| cm1672 |
| cm2bb |
| cm8bb |
| cm10bb |
| cm161bb |
| BKO_1037 |
| SGO_08 |
| SGO_27 |
| SGO_47 |
| SGO_71 |
| SGO_86 |
| ARV26 |
| CHU12 |
| HXB2-LAI-IIIB-BRU |
| 445_124360B |
| UG031 |
| UG029 |
| 92RW008 |
| 92UG037 |
| ETH_G_079 |
| 92RW025A |
| 93RW037A |
| UG275 |
| Q23_17 |
| 94RW13 |
| 97TZ02_AF361872 |
| 97TZ03_AF361873 |
| 113913U |
| 115106J |
| 123208J |
| 123793K |
| 123898B |
| 125636U |
| 126533T |
| 130526D |
| 132074M |
| 132318B |
| 132591A |
| 132685M |
| 134248P |
| 136677C |
| 137499Q |
| 138162O |
| 138430V |
| 138904N |
| 139177O |
| 140727Y |
| 141601A |
| 142181Q |
| 144449S |
| 145027T |
| 145484O |
| 146922V |
| 147521R |
| 148838K |
| 150014T |
| 151848F |
| KER2008 |
| KER2009 |
| KER2012 |
| KNH1088 |
| KNH1135 |
| KNH1144 |
| KNH1199 |
| KNH1207 |
| KNH1209 |
| KNH1211 |
| KSM4021 |
| KSM4024 |
| KSM4030 |
| MSA4069 |
| MSA4070 |
| MSA4072 |
| MSA4076 |
| MSA4079 |
| NKU3005 |
| 99UGA07072 |
| 99UGG03379 |
| 98UG57134 |
| 98UG57135 |
| 98UG57136 |
| 98UG57142 |
| ML170_1986 |
| 98SN_15HALD |
| 98SN-69HPD |
| 99SN_61HPD_15_10_99 |
| 98SN_5hald_20_7_98 |
| CK2_75 |
| CK22_50 |
| MKK248 |
| SU478 |
| SU238 |
| 119758j_185 |
| 124727i_164 |
| 97CIRMF21 |
| 22 |
| 54 |
| A173 |
| A341 |
| ML013_10 |
| ML013_2 |
| ML605_3 |
| ML752 |
| 667 |
| 850 |
| 842 |
| 22 |
| 45 |
| 51 |
| 185 |
| 164 |
| 180 |
| 401 |
| 429 |
| 453 |
| 485 |
| 545 |
| 584 |
| 534 |
| 532 |
| 604 |
| 623 |
| 599 |
| 596 |
| 667 |
| 652 |
| 704 |
| 687 |
| 801 |
| 742 |
| 790 |
| 807 |
| 816 |
| 841 |
| 847 |
| 850 |
| 879 |
| 871 |
| 14 |
| 4 |
| 31 |
| 46 |
| 34 |
| 32 |
| 57 |
| 85 |
| 69 |
| 58 |
| 157 |
| 97 |
| 192 |
| 225 |
| 175 |
| 162 |
| 252 |
| 204 |
| 209 |
| 231 |
| 199 |
| 228 |
| 221 |
| 253 |
| 408 |
| 421 |
| 407 |
| 460 |
| 439 |
| 446 |
| 415 |
| 451 |
| 403 |
| 508 |
| 437 |
| 458 |
| 488 |
| 514 |
| 445 |
| 427 |
| 487 |
| 520 |
| 551 |
| 576 |
| 560 |
| 589 |
| 651 |
| 562 |
| 572 |
| 582 |
| 573 |
| 635 |
| 614 |
| 619 |
| 659 |
| 661 |
| 662 |
| 697 |
| 701 |
| 686 |
| 679 |
| 684 |
| 580 |
| 672 |
| 674 |
| 690 |
| 725 |
| 737 |
| 731 |
| 507 |
| 761 |
| 754 |
| 757 |
| 744 |
| 771 |
| 726 |
| 776 |
| 798 |
| 820 |
| 821 |
| 838 |
| 845 |
| 811 |
| 819 |
| 866 |
| 839 |
| 877 |
| 861 |
| 869 |
| 881 |
| 882 |
| A_122_335 |
| A_140_223 |
| A_161_287 |
| A_194_793 |
| A_225_706 |
| A_264_615 |
| A_264_643 |
| A_326_642 |
| A_ABB08 |
| A_ABB102 |
| A_ABB165 |
| A_ABB213 |
| 97KE104 |
| 97KE173 |
| 98KE178 |
| 99KE432 |
| 96KE449 |
| 00KE561 |
| 99KE579 |
| 02ZA_DR17 |
| 93RW_024 |
| 92UG_029 |
| 111186L |
| 111740R |
| 111976H |
| 112979I |
| 114892W |
| 121635E |
| 121766I |
| 121874N |
| 122286N |
| 122530R |
| 122742D |
| 122744X |
| 123114S |
| 123768V |
| 126151S |
| 126721B |
| 126951L |
| 128124P |
| 128315G |
| 128375T |
| 129324I |
| 132590N |
| 133395E |
| 133592I |
| 133725L |
| 135274M |
| 136075T |
| 136579G |
| 136668K |
| 137067Y |
| 138829X |
| 139277H |
| 139330R |
| 141070V |
| 141906R |
| 142114X |
| 142425V |
| 144857P |
| 144963H |
| 145058J |
| 146342V |
| 147272W |
| 147418O |
| 147572M |
| 148095P |
| 148639N |
| 148882I |
| 149254I |
| 149594A |
| 150205H |
| KER2008_patent |
| TC029004 |
| TC026104 |
| TC025204 |
| TC024604 |
| TC023504 |
| TC022504 |
| TC022404 |
| TC022204 |
| TC021904 |
| TC021704 |
| TC021404 |
| TC021204 |
| TC020504 |
| TC011303 |
| TC010603 |
| TC009903 |
| TC009003 |
| TC008903 |
| TC007803 |
| QC890 |
| QB424 |
| QB596 |
| QB670 |
| 04ZASK162B1 |
| CM_01_133 |
| CM_01_568 |
| P0034_090205 |
| P0054_020305 |
| P0060_070305 |
| P0079_160305 |
| P0110_120405 |
| P0185_130605 |
| P0218_130705 |
| P0289_140905 |
| MBA1021_170805 |
| MBA1025_180805 |
| MBA1028_230805 |
| MBA1037_060905 |
| MBA1053_201005 |
| MBAR005_120405 |
| MBAR007_120405 |
| MBAR016_020505 |
| MBAR017_030505 |
| 004_106665H |
| 014_108422Z |
| 022_109985S |
| 031_106897U |
| 032_109180U |
| 034_109734M |
| 044_110390I |
| 045_109688N |
| 046_110300R |
| 051_110961Y |
| 057_110308G |
| 097_117239C |
| 180_118080i |
| 199_114660F |
| 225_119778C |
| 401_120082s |
| 403_123871S |
| 407_124279Q |
| 408_125049Q |
| 421_125042G |
| 427_125290E |
| 429_125330t |
| 439_126157P |
| 440_126149o |
| 446_126268V |
| 458_126651R |
| 466_124362l |
| 476_126649x |
| 485_127960U |
| 487_126068K |
| 496_127964E |
| 503_127844l |
| 508_128740N |
| 514_128712J |
| 522_128205c |
| 534_129066r |
| 542_129863t |
| 551_129563G |
| 560_129773M |
| 562_128605K |
| 572_131039B |
| 574_130458m |
| 576_131187D |
| 577_131301I |
| 580_130747L |
| 582_130283Q |
| 584_131489e |
| 592_129775v |
| 608_131002r |
| 614_133371R |
| 622_133749h |
| 623_134023j |
| 635_134512Y |
| 638_134023j |
| 646_133038m |
| 649_134513d |
| 651_135200J |
| 652_133291l |
| 653_134024g |
| 661_135234B |
| 662_135975N |
| 667_136472S |
| 672_135604H |
| 674_137072A |
| 679_137708Q |
| 684_134386Y |
| 686_137213K |
| 690_138008N |
| 697_138073B |
| 699_136283t |
| 701_138581J |
| 702_136536A |
| 704_138427k |
| 725_138071W |
| 726_138858J |
| 731_138858J |
| 734_140160a |
| 737_140207H |
| 742_139543x |
| 744_140410S |
| 754_140838E |
| 757_139187G |
| 141262J |
| 771_140407R |
| 776_141841G |
| 792_142856n |
| 798_141839U |
| 801_143188S |
| 802_142555O |
| 805_143343E |
| 807_141911f |
| 811_145165P |
| 819_144897T |
| 820_144896A |
| 821_144894D |
| 838_145431G |
| 839_146276F |
| 841_146190g |
| 842_144505j |
| 850_145864u |
| 861_146980X |
| 866_147223K |
| 869_147440T |
| 871_146977m |
| 876_147573X |
| 877_147575W |
| 879_147225s |
| 881_147226R |
| 117948S |
| 129779W |
| 133750R |
| 139830I |
| ML752 |
| ML1901 |
| ML1945 |
| ML1990 |
| ML2014 |
| ML1901PCR2 |
| ML1990PCR |
| GH_007 |
| GH_142 |
| GH_182 |
| GH_243 |
| GH_244 |
| TBK002 |
| TBK004 |
| TBK005 |
| TBK011 |
| TBK017 |
| TBK018 |
| TBK023 |
| TBK024 |
| TBK037 |
| TBK039 |
| TBK040 |
| TBK045 |
| TBK048 |
| TBK056 |
| TBK060 |
| TBK064 |
| TBK067 |
| TBK090 |
| TBK095 |
| TBK099 |
| TMS001 |
| TMS002 |
| TMS006 |
| TMS007 |
| TMS011 |
| TMS016 |
| TMS018 |
| TMS025 |
| TMS103 |
| TMS108 |
| TMS115 |
| TMS121 |
| TMS123 |
| TMS124 |
| TMS127 |
| TMS131 |
| TMS134 |
| TMS137 |
| TMS141 |
| TMS142 |
| TMS147 |
| TMS206 |
| TMS218 |
| TMS301 |
| TMS305 |
| TMS306 |
| TMS314 |
| TMS319 |
| TMS320 |
| TMS336 |
| TMS337 |
| TMS341 |
| TMS343 |
| IAEAM003 |
| IAEAM004 |
| IAEAM007 |
| IAEAM012 |
| IAEAM013 |
| IAEAM014 |
| IAEM017 |
| IAEAM019 |
| IAEM022 |
| IAEM028 |
| IAEM031 |
| IAEM033 |
| IAEM038 |
| IAEM039 |
| IAEM041 |
| IAEM043 |
| IAEM045 |
| IAEM049 |
| IAEM050 |
| IAEM054 |
| IAEM056 |
| IAEM060 |
| IAEM065 |
| IAEM067 |
| IAEM078 |
| BKO_902 |
| BKO_1023 |
| BKO_1047 |
| SGO_49 |
| 335346R |
| 341741Y |
| 349844H |
| 361358A |
| 362363H |
| 363290D |
| 326163_M |
| 365770P |
| 366975Y |
| 367927_J |
| 369096E |
| 377986_M |
| 380521_H |
| 387519_L |
| 390900V |
| 394901B |
| 400177E |
| 400196E |
| 409924L |
| 413282X |
| 413433L |
| 415189_B |
| 417659J |
| 430066A |
| 460471T |
| 476384H |
| 289005 |
| SK162 |
| 07_156895 |
| 07_156927 |
| 07_156928 |
| 07_156929 |
| 07_156944 |
| 07_156948 |
| 07_156956 |
| 07_156961 |
| 07_156967 |
| 07_156976 |
| 07_156979 |
| 07_156997 |
| 07_156998 |
| 07_157001 |
| 08_101504 |
| 08_101507 |
| 08_101510 |
| 08_101518 |
| 08_101521 |
| 08_101544 |
| 08_101577 |
| 08_102864 |
| 08_102868 |
| 08_102902 |
| TR178 |
| TR185 |
| TR257 |
| 06KECst_006 |
| 06KECst_007 |
| 06KECst_021 |
| 06KECst_019 |
| 06KECst_028 |
| 06KECst_009 |
| 06KECst_005 |
| 06KECst_020 |
| 06KECst_016 |
| 06KECst_022 |
| 06KECst_013 |
| 06KECst_025 |
| 06KECst_001 |
| 06KECst_017 |
| TV239 |
| TV314 |
| Cameroon_20 |
| Cameroon_55 |
| ZZP625 |
| ZZP628 |
| ZZP649 |
| ZZP671 |
| ZZP877 |
| ZZP963 |
| ZZQ675 |
| ZZQ946 |
| ZZR40 |
| ZZQ955 |
| ZZQ956 |
| ZZR160 |
| ZZR475 |
| 06TGHT045 |
| 06TGHT055 |
| 06TGHT077 |
| 06TGHT085 |
| 06TGHT126 |
| 08GQ182 |
| 08GQ508 |
| 08GQ281 |
| 114HALD |
| 337HALD |
| 61HPD |
| 935hald |
| 1700_hald |
| A1_07CD1246 |
| A1_07CD0509 |
| A1_07CD0444 |
| A1_07CD3665 |
| A_07CD3012 |
| A_07CD3729 |
| URF_A_CRF22_07CD3407 |
| A_07CD0050 |
| A_07CD2110 |
| A_07CD4292 |
| A_07CD1425 |
| A_07CD1525 |
| URF_A_A3_A_07CD2084 |
| A_07CD3373 |
| A1_07CD3784 |
| A_07CD0335 |
| A_07CD0040 |
| A1_07CD2974 |
| A_07CD2657 |
| A_07CD3537 |
| A_07CD3191 |
| A_07CD4078 |
| 13020D_P1 |
| 13228D_P1 |
| 13240D_P1 |
| 13263D_P1 |
| 13305D_P1 |
| 13354D_P1 |
| 13929D_P1 |
| 14006D_P1 |
| 14275D_P1 |
| 14324D_P1 |
| 14411D_P1 |
| 14493D_P1 |
| 14888D_P1 |
| 15017D_P1 |
| 15460D_P1 |
| 15545D_P1 |
| 15710D_P1 |
| 15712D_P1 |
| 15911D_P1 |
| 16079D_P1 |
| 16182D_P1 |
| 16455D_P1 |
| 16712D_P1 |
| 17219D_P1 |
| DT0016_05_P1 |
| DT0971_05_P1 |
| DT1515_04_P1 |
| DT1745_05_P1 |
| DT2172_05_P1 |
| DT2373_05_P1 |
| DT2794_05_P1 |
| DT2829_05_P1 |
| DT2986_05_P1 |
| DT3165_05_P1 |
| DT3282_05_P1 |
| DT3441_05_P1 |
| DT3482_05_P1 |
| DT3513_05_P1 |
| DT3516_05_P1 |
| DT3736_05_P1 |
| DT3793_05_P1 |
| DT3994_05_P1 |
| DT4014_05_P1 |
| DT3340_05_P1 |
| 301V03000895 |
| 301V03000981BM |
| 301V03000985 |
| 301V04000057BM |
| 301V04000184 |
| 301V04000665BM |
| 301V04000684 |
| 301V04000864 |
| 301V04000900BM |
| 301V04000911BM |
| 301V04002129 |
| 301V04002157BM |
| 301V04002594 |
| 301V04002668BM |
| 301V04003339BM |
| 301V04003358 |
| 301V04003475BM |
| 301V04003479 |
| 301V04003733 |
| 301V04003895 |
| 301V04003959BM |
| 301V04004030BM |
| 301V04004298 |
| 301V04004337BM |
| 301V04004694 |
| 301V04004804BM |
| 301V04005655 |
| 301V04005698BM |
| 301V04005942 |
| 301V04005975BM |
| CYV75 |
| CYR20 |
| CYV085 |
| CYR028 |
| CYN851 |
| CYN732 |
| CDL80 |
| SRCDD0242 |
| HG_0857 |
| HG_0943 |
| HG_0130 |
| HU_0017 |
| SK_0004 |
| CK1_1 |
| CK1_20 |
| CK1_46 |
| CK1_45 |
| CK1_40 |
| CK1_25 |
| CK1_47 |
| VP_LT_111 |
| VP_SW_2 |
| VP_SW_1 |
| VP_SW_11 |
| ORAV279 |
| ORAV280 |
| ORAV271 |
| 07ZA17579 |
| CM04_073 |
| CM04_094 |
| CM04_096 |
| CM04_064 |
| SN756HALD |
| SN826HALD |
| SN2720HALD |
| 320024_A |
| 320208_V |
| 322176_A |
| 337194_A |
| 337314_Y |
| 337373_S |
| 338325_D |
| 341569_X |
| 341815_K |
| 348121_H |
| 350052_R |
| 384132_R |
| 436546_P |
| 436547_D |
| 439208_K |
| 439552_J |
| 440672_L |
| 317296_E |
| 318244_S |
| 318883_Z |
| 319763_R |
| 320477_T |
| 321288_A |
| 321541_D |
| 326455_J |
| 328958_W |
| 332768_D |
| 337081_S |
| 338135_K |
| 338796_K |
| 341279_H |
| 362487_R |
| 370903_R |
| 371850_K |
| 385463_H |
| 385494_T |
| 393958_V |
| 404615_W |
| 413614_D |
| 427986_C |
| 430253_Z |
| 436063_G |
| 393250_S |
| 393405_H |
| 416275_M |
| 425113_S |
| 431730_B |
| 519810_R |
| 521684_A |
| 529392_J |
| 482043_M |
| 503727_G |
| 504080_C |
| 509635_D |
| 516673_E |
| CP076300084 |
| CP076300141 |
| CP076300688 |
| 1_0212_KiBS_V7 |
| 1_066_KiBS_V5 |
| 1_0517_KiBS_V5 |
| 1_0360_KiBS_V3 |
| 1_0472_KiBS_V5 |
| 1_0457_KiBS_V3 |
| 1_0496_6_KiBS_V3 |
| 0_0158_KiBS_V16 |
| 0_0472_KiBS_V16 |
| 1_0437_KiBS_V5 |
| 1_0357_KiBS_V5 |
| 1_0289_KiBS_V5 |
| TZ_10_002026_A1 |
| TZ_08_014497_A1 |
| TZ_09_032657_A1 |
| TZ_09_006010_A1 |
| TZ_09_032639_A1 |
| TZ_10_003313_A1 |
| TZ_09_001336_A1 |
| TZ_08_017180_A1 |
| TZ_08_022112_A1 |
| TZ_08_014496_A1 |
| TZ_08_023282_A1 |
| TZ_08_017177_A1 |
| TZ_08_017190_A1 |
| TZ_10_002029_A1 |
| TZ_08_023280_A1 |
| TZ_08_017183_A1 |
| TZ_10_003320_A1 |
| TZ_10_003309_A1 |
| TZ_09_032646_A1 |
| TZ_09_006021_A1 |
| TZ_10_003314_A1 |
| TZ_08_017174_A1 |
| TZ_08_023285_A1 |
| TZ_09_006015_A1 |
| TZ_08_017187_A1 |
| TZ_08_017178_A1 |
| TZ_09_032655_A1 |
| TZ_09_001326_A1 |
| TZ_08_029745_A1 |
| C435S_J0 |
| C455S_J0 |
| D344T_J0 |
| D375T_J0 |
| NTCPS729 |
| AICPS434 |
| AICPS399 |
| NTCPS726 |
| NTCPS724 |
| NTCPS720 |
| NTCPS719 |
| NTCPS731 |
| NTCPS717 |
| NTCPS716 |
| AICPS335 |
| AICPS107 |
| AICPS435 |
| AICPS438 |
| AICPS495 |
| AICPS432 |
| NTCPS733 |
| AICPS157 |
| AICPS336 |
| AICPS303 |
| AICPS283 |
| AICPS225 |
| AICPS216 |
| AICPS180 |
| NTCPS709 |
| NTCPS707 |
| NTCPS702 |
| NTCPS701 |
| AICPS165 |
| AICPS158 |
| AICPS106 |
| AICPS005 |
| AICPS001 |
| AICPS006 |
| AICPS009 |
| AICPS035 |
| UGNTC737 |
| 07NG_SN022 |
| 07NG_SN305 |
| 07NG_SN223 |
| 07NG_SN077 |
| 07NG_SN277 |
| 07NG_SN132 |
| 07NG_SN157 |
| SK6031_M18 |
| SK6039_M24 |
| SK9037_PI |
| SK6074_PI |
| SK3015_PI |
| SK8040_PI |
| SK5010_PI |
| SK9027_PI |
| SK5013_PI |
| 0765HR |
| 2917HR |
| 3132HR |
| 3272HR |
| 3445HR |
| 4668HR |
| 6356HR |
| 6699HR |
| 7475HR |
| 7518HR |
| 7672HR |
| 3083HR |
| 4834HR |
| 3153HR |
| 3635HR |
| 3683HR |
| 6891HR |
| U455_U455A |
| 97CDKFE4 |
| 02CD_KBS187 |
| 02CD_KSS004 |
| A2_225_692 |
| QA779 |
| QA284 |
| 01CM_1445MV |
| P32_16_1a10 |
| P32_16_1b3 |
| P32_16_1b4 |
| P32_16_1b5 |
| P32_16_1b7 |
| P32_16_1c2 |
| P32_16_1c5 |
| P32_16_1c7 |
| P32_16_1e5 |
| P32_16_1f1 |
| P32_16_1f3 |
| P32_16_1f2 |
| P32_16_2a6 |
| P32_16_2b3 |
| P32_16_2c7 |
| P32_16_2c8 |
| P32_16_2d2 |
| P32_16_2d6 |
| P32_16_2e1 |
| P32_16_2e9 |
| P32_16_4a2 |
| P32_16_4a4 |
| P32_16_4b6 |
| P32_16_4b7 |
| P32_16_4b11 |
| P32_16_4c12 |
| P32_16_4d4 |
| P32_16_4d6 |
| P32_16_4d7 |
| P32_16_4e6 |
| P32_16_4e8 |
| P32_16_4f4 |
| P32_24_2e7 |
| P32_24_1a3 |
| P32_24_1a5 |
| P32_24_1a8 |
| P32_24_1b9 |
| P32_24_1c7 |
| P32_24_1c8 |
| P32_24_1e8 |
| P32_24_2a10 |
| P32_24_2b7 |
| P32_24_2b12 |
| P32_24_2c6 |
| P32_24_2e2 |
| P32_24_2e4 |
| P32_24_2f6 |
| P32_24_2f9 |
| P32_24_3a2 |
| P32_24_3a8 |
| P32_24_3d4 |
| P32_24_3d11 |
| P32_24_3e9 |
| P32_24_3f8 |
| P32_24_4a3 |
| P32_24_4a4 |
| P32_24_4a12 |
| P32_24_4b5 |
| P32_24_4b9 |
| P32_24_4b10 |
| P32_24_4c4 |
| P32_24_4c12 |
| P32_24_4f8 |
| P32_44_1a8 |
| P32_44_2a3 |
| P32_44_2a8 |
| P32_44_2c8 |
| P32_44_2d3 |
| P32_44_3a1 |
| P32_44_3a4 |
| P32_44_3a12 |
| P32_44_3c5 |
| P32_44_3d3 |
| P32_44_3f8 |
| P32_44_3f10 |
| 71377PL10 |
| A57 |
| A58 |
| A41 |
| A44 |
| A46 |
| A51 |
| A55 |
| A56 |
| A63 |
| A69 |
| A71 |
| A72 |
| A52 |
| A54 |
| A42 |
| A43 |
| A62 |
| A5 |
| A3 |
| A61 |
| A65 |
| A21 |
| A26 |
| A31 |
| A19 |
| A25 |
| A28 |
| A29 |
| A70 |
| A73 |
| A33 |
| A22 |
| A24 |
| A32 |
| A39 |
| A40 |
| A48 |
| 94CY017_41 |
| CY008 |
| CY009 |
| CY012 |
| CY015 |
| CY057 |
| CY058 |
| CY111 |
| CY112 |
| CY153 |
| CY169 |
| CY170 |
| CY171 |
| CY173 |
| CY178 |
| CY021 |
| CY022 |
| CY023 |
| CY051 |
| CY064 |
| CY106 |
| CY121 |
| CY140 |
| CY164 |
| CY182 |
| CY185 |
| CY196 |
| CY207 |
| CY208 |
| CY209 |
| CY213 |
| CY218 |
| CY230 |
| CY231 |
| CY235 |
| CY236 |
| CY243 |
| CY255 |
| 67429PL12 |
| 85344PL1 |
| 71264PL11 |
| 87516PL1 |
| 89629PL1 |
| 89794PL1 |
| 71372PL14 |
| 75925PL8 |
| 82472PL1 |
| 75925PL9 |
| SL17852PL1 |
| 94275PL1 |
| 95074PL1 |
| 95310PL1 |
| 94412PL1 |
| 95314PL1 |
| 94412PL2 |
| 94389PL1 |
| 75925PL10 |
| 97023PL1 |
| 94412PL3 |
| 88882PL1 |
| 88662PL1 |
| 88372PL1 |
| 89160PL1 |
| 89161PL1 |
| 88518PL1 |
| 88723PL1 |
| 98821PL1 |
| 75925PL11 |
| 99255PL1 |
| 99517PL1 |
| 99925PL1 |
| 99772PL1 |
| 100614PL1 |
| 72193PL19 |
| 102032PL1 |
| 94412PL5 |
| 101297PL1 |
| 75925PL12 |
| 102766PL1 |
| 102620PL1 |
| 94412PL6 |
| 89160PL3 |
| 104198PL1 |
| 104100PL1 |
| 103562PL1 |
| 103995PL1 |
| 103880PL1 |
| 104103PL1 |
| 103887PL1 |
| 104589PL1 |
| 94412PL8 |
| 76153PL5 |
| 105739PL1 |
| 71264PL12 |
| 98821PL2 |
| 106804PL1 |
| 94412PL10 |
| 75925PL14 |
| 108393PL1 |
| 109856PL1 |
| 76153PL6 |
| 111637PL1 |
| 111640PL1 |
| 112522PL1 |
| 112688PL1 |
| 113058PL1 |
| 114213PL1 |
| 113727PL1 |
| 114099PL1 |
| 114942PL1 |
| 115520PL1 |
| 115989PL1 |
| 76153PL7 |
| 116508PL1 |
| 116214PL1 |
| 116928PL1 |
| 116880PL1 |
| 117167PL1 |
| 117345PL1 |
| 111863PL1 |
| 117052PL1 |
| 107836PL4 |
| 119984PL1 |
| 117058PL1 |
| 120094PL1 |
| 107836PL6 |
| 121001PL1 |
| 71164PL14 |
| 122395PL1 |
| 121653PL1 |
| 122644PL1 |
| SI4037_02 |

**Dataset B**

| EU345825 |
| --- |
| EU345824 |
| EU345823 |
| EU345822 |
| EU345821 |
| EU345820 |
| EU345819 |
| EU345818 |
| EU345817 |
| EU345815 |
| EU345803 |
| EU345802 |
| EU345801 |
| EU345800 |
| EU345799 |
| EU345773 |
| EU345771 |
| EU345770 |
| EU345767 |
| EU345766 |
| EU345765 |
| EU345763 |
| EU345762 |
| EU345761 |
| EU345760 |
| EU345722 |
| EU345721 |
| EU345719 |
| EU345718 |
| EU345716 |
| EU345715 |
| EU345714 |
| EU345713 |
| EU345711 |
| EU345709 |
| EU345905 |
| EU345904 |
| EU345903 |
| EU345902 |
| EU345901 |
| EU345900 |
| EU345899 |
| EU345898 |
| EU345897 |
| EU345895 |
| EF545110 |
| EF545109 |
| AY500393 |
| FJ864679 |
| FJ822134 |
| FJ822133 |
| FJ822132 |
| FJ822131 |
| FJ822130 |
| FJ822129 |
| FJ822128 |
| FJ822127 |
| FJ822126 |
| FJ009813 |
| FJ009812 |
| FJ009811 |
| FJ009809 |
| FJ009808 |
| FJ009807 |
| FJ009806 |
| FJ009805 |
| FJ009804 |
| FJ009803 |
| GQ867635 |
| GQ867631 |
| GQ867630 |
| GQ867628 |
| GQ867627 |
| GQ867625 |
| GQ867624 |
| HQ161930 |
| HQ161929 |
| HQ161928 |
| HQ161927 |
| HQ161926 |
| HQ161925 |
| HQ161924 |
| HQ161923 |
| HQ161921 |
| HQ130000 |
| HQ129999 |
| HQ129998 |
| HQ129997 |
| HQ129996 |
| HQ129995 |
| HQ129994 |
| HQ129993 |
| HQ129991 |
| HQ129990 |
| HQ129989 |
| HQ449403 |
| HQ449402 |
| HQ449398 |
| HQ449397 |
| HQ449395 |
| HQ449393 |
| HQ896679 |
| HM345560 |
| HM345558 |
| HM345554 |
| HM345536 |
| HM345579 |
| HM345575 |
| HM345569 |
| HM345565 |
| HM345553 |
| HM345551 |
| HM345539 |
| HM345537 |
| JN673405 |
| JQ292898 |
| JQ292896 |
| JQ292894 |
| JQ292899 |
| JQ292895 |
| JQ292893 |
| JX141227 |
| JX141225 |
| JX141223 |
| JX141221 |
| JX141219 |
| JX141217 |
| JX141215 |
| JX141213 |
| JX141211 |
| JX141209 |
| JX141226 |
| JX141224 |
| JX141222 |
| JX141220 |
| JX290238 |
| JX290236 |
| JX290234 |
| JX290232 |
| JX290230 |
| JX290228 |
| JX290226 |
| JX290224 |
| JX290222 |
| JX290218 |
| JX500694 |
| KC156532 |
| KC156533 |
| KC254612 |
| KC254643 |
| KC254641 |
| KC254639 |
| KC254637 |
| KC254635 |
| KC254633 |
| KC254631 |
| KC665939 |
| KC665938 |
| KC665937 |
| KC665936 |
| KC665934 |
| KC665933 |
| KC665932 |
| KC665931 |
| KC665930 |
| KC665929 |
| KC665928 |
| KF257884 |
| KF257882 |
| KF257881 |
| KF257880 |
| KF257879 |
| KF257878 |
| KF257877 |
| KF257876 |
| KF257875 |
| KF257874 |
| KF257873 |
| KF257870 |
| KF374888 |
| KF374887 |
| KF374886 |
| KF374885 |
| KF374884 |
| KF374883 |
| KF374882 |
| KF374881 |
| KF374880 |
| KF374879 |
| KF374878 |
| KF374877 |
| KF374876 |
| KF374875 |
| KF374872 |
| KF374871 |
| KF374870 |
| KF374869 |
| KF374868 |
| KF374867 |
| KF374866 |
| KF374865 |
| KF374864 |
| KF374863 |
| KF374862 |
| KF374861 |
| KF374860 |
| KF374859 |
| KF374858 |
| KF374857 |
| KF374856 |
| KF374855 |
| KF374854 |
| KF374853 |
| KF374852 |
| KF374851 |
| KF971945 |
| KF971938 |
| KF971937 |
| KF971936 |
| KF971933 |
| KF971929 |
| KF971926 |
| KF971920 |
| KF971919 |
| KJ499635 |
| KJ499634 |
| KJ499633 |
| KJ499632 |
| KJ499631 |
| KJ499630 |
| KJ499628 |
| KJ499626 |
| KJ499625 |
| KJ499622 |
| KJ499621 |
| KJ499620 |
| KJ499619 |
| KJ499618 |
| KJ499617 |
| KJ499616 |
| KJ499615 |
| KJ499614 |
| KJ499612 |
| KJ499611 |
| KJ499610 |
| KJ499609 |
| KJ499592 |
| KJ499591 |
| KJ499590 |
| EU345703 |
| EU345738 |
| FJ009725 |
| FJ009731 |
| FJ009736 |
| FJ009750 |
| FJ009751 |
| FJ009754 |
| FJ009768 |
| FJ009774 |
| FJ822080 |
| KF716492 |
| KF716491 |
| JX500695 |
| JX500696 |
| KJ499563 |
| KJ499562 |
| KJ499561 |
| KJ499560 |
| KJ499559 |
| KJ722123 |
| KJ722122 |
| KJ722121 |
| KJ722120 |
| KJ722119 |
| KJ722118 |
| KJ722117 |
| KJ722116 |
| KJ722115 |
| KJ722114 |
| KJ722113 |
| KJ722109 |
| KJ722075 |
| KJ722074 |
| KJ722073 |
| KJ722072 |
| KJ722071 |
| KJ722070 |
| KJ197223 |
| KJ197220 |
| KJ197218 |
| EU345840 |
| EU345839 |
| EU345838 |
| EU345837 |
| EU345836 |
| EU345835 |
| EU345834 |
| EU345833 |
| EU345832 |
| EU345831 |
| DQ823367 |
| DQ823366 |
| DQ823365 |
| DQ823361 |
| DQ823360 |
| DQ823359 |
| DQ823358 |
| DQ823357 |
| DQ823356 |
| DQ055315 |
| DQ055313 |
| DQ055310 |
| DQ055308 |
| DQ055307 |
| DQ055306 |
| DQ055304 |
| DQ055303 |
| DQ055302 |
| DQ055301 |
| DQ055299 |
| DQ055298 |
| DQ055297 |
| DQ055295 |
| DQ055293 |
| DQ055291 |
| DQ055290 |
| DQ055289 |
| DQ055288 |
| DQ055285 |
| DQ055283 |
| DQ055282 |
| DQ055281 |
| DQ055279 |
| DQ055278 |
| DQ055277 |
| DQ055276 |
| DQ055274 |
| DQ055273 |
| DQ055272 |
| DQ055271 |
| DQ055270 |
| DQ055269 |
| DQ055268 |
| DQ055267 |
| DQ055266 |
| DQ055265 |
| DQ055264 |
| DQ055263 |
| DQ055262 |
| DQ055261 |
| DQ055260 |
| DQ055259 |
| DQ055257 |
| DQ055256 |
| DQ055255 |
| DQ055254 |
| DQ055253 |
| DQ055252 |
| DQ055251 |
| DQ055248 |
| DQ055247 |
| DQ055246 |
| DQ055245 |
| DQ055244 |
| DQ055243 |
| DQ055242 |
| DQ055241 |
| DQ055240 |
| DQ055239 |
| DQ055238 |
| DQ055235 |
| DQ055234 |
| DQ055233 |
| DQ055232 |
| DQ055231 |
| DQ055230 |
| DQ055229 |
| DQ055225 |
| DQ055224 |
| DQ055223 |
| DQ055221 |
| DQ055219 |
| DQ055210 |
| DQ055206 |
| DQ055204 |
| DQ055199 |
| DQ055196 |
| DQ055195 |
| DQ055194 |
| DQ055193 |
| DQ055192 |
| DQ055191 |
| DQ055190 |
| DQ055189 |
| DQ055188 |
| DQ055187 |
| DQ055186 |
| DQ055184 |
| DQ055183 |
| DQ055182 |
| DQ055181 |
| DQ055180 |
| DQ055178 |
| DQ055177 |
| DQ055176 |
| DQ055175 |
| DQ055174 |
| AF413987 |
| HQ161910 |
| HQ161909 |
| HQ161908 |
| HQ161907 |
| HQ161906 |
| HQ115075 |
| HQ115071 |
| HQ115070 |
| HQ115068 |
| HQ115067 |

**Dataset C**

| DQ823367 |
| --- |
| DQ823366 |
| DQ823365 |
| DQ823361 |
| DQ823360 |
| DQ823359 |
| DQ823358 |
| DQ823357 |
| DQ823356 |
| AF413987 |
| AF025596 |
| AF025595 |
| AF025594 |
| AF025593 |
| AF025592 |
| AF025591 |
| AF025590 |
| AF025589 |
| AF025588 |
| AF025587 |
| AF025586 |
| AF025585 |
| AF025584 |
| AF025583 |
| AF025582 |
| AF025581 |
| AF025580 |
| AF082486 |
| U93676 |
| U93665 |
| AF100939 |
| AF100934 |
| AF098953 |
| EF545108 |
| AY500393 |
| FJ864679 |
| GU481683 |
| GU481660 |
| GU481635 |
| GU481589 |
| GU481565 |
| GU481536 |
| GU481518 |
| GU481471 |
| GU481420 |
| GU481391 |
| GU481370 |
| GU481328 |
| GU481276 |
| GU481253 |
| GU481202 |
| GU481155 |
| HQ385842 |
| HQ385840 |
| HQ385839 |
| HQ385838 |
| HQ385837 |
| HQ385830 |
| HQ385820 |
| HQ385819 |
| HQ385818 |
| HQ385817 |
| HQ338117 |
| HQ338113 |
| HQ338112 |
| HQ338111 |
| HQ616096 |
| HQ616092 |
| HQ616084 |
| HQ616085 |
| HQ616083 |
| JF327807 |
| JF430896 |
| JQ292900 |
| JQ292898 |
| JQ292896 |
| JQ292892 |
| JQ292897 |
| JQ292895 |
| HQ834965 |
| HQ896492 |
| HQ896489 |
| JF952017 |
| JF952015 |
| JF952013 |
| JF952018 |
| JF952016 |
| JF952014 |
| JF952012 |
| JX500695 |
| JX500696 |
| JX500694 |
